# Singlet and Triplet Pathways Determine the Thermal *Z*/*E* Isomerization of an Arylazopyrazole-Based Photoswitch

**DOI:** 10.1021/acs.jpclett.3c01785

**Published:** 2023-09-29

**Authors:** Nadja K. Singer, Katharina Schlögl, J. Patrick Zobel, Marko D. Mihovilovic, Leticia González

**Affiliations:** † Institute of Theoretical Chemistry, Faculty of Chemistry, University of Vienna, Währinger Str. 17, 1090 Vienna, Austria; ‡ Vienna Doctoral School in Chemistry (DoSChem), University of Vienna, Währinger Str. 42, 1090 Vienna, Austria; ¶ Institute of Applied Synthetic Chemistry, TU Wien, Getreidemarkt 9, 1060 Vienna, Austria

## Abstract

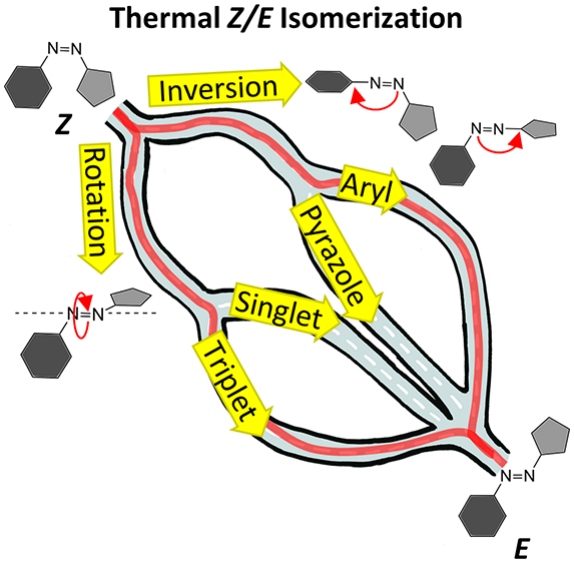

Understanding the thermal isomerization mechanism of azobenzene derivatives is essential to designing photoswitches with tunable half-lives. Herein, we employ quantum chemical calculations, nonadiabatic transition state theory, and photosensitized experiments to unravel the thermal *Z*/*E* isomerization of a heteroaromatic azoswitch, the phenylazo-1,3,5-trimethylpyrazole. In contrast to the parent azobenzene, we predict two pathways to be operative at room temperature. One is a conventional ground-state reaction occurring via inversion of the aryl group, and the other is a nonadiabatic process involving intersystem crossing to the lowest-lying triplet state and back to the ground state, accompanied by a torsional motion around the azo bond. Our results illustrate that the fastest reaction rate is not controlled by the mechanism involving the lowest activation energy, but the size of the spin–orbit couplings at the crossing between the singlet and the triplet potential energy surfaces is also determinant. It is therefore mandatory to consider all of the multiple reaction pathways in azoswitches in order to predict experimental half-lives.

Photoswitches are light-responsive compounds that can interconvert reversibly between two structures. Isomerization from the most stable one to the least stable conformer takes place upon light irradiation, while the initial structure can be restored by light or thermal activation. Photoswitches are widely exploited as smart materials in a number of applications^1−5^ but also as drugs in synthetic photobiology or photopharmacology.^6−11^ For the latter, they are attached to a target bioactive compound, meaning that additional drug-design criteria must be met. An ideal photoswitch in photopharmacology should not induce toxicity, be robust over many cycles, absorb preferably in the therapeutic near-infrared window, show high efficiency in the photoisomerization process, and result in an on–off change of activity on the biological target.^8,9^ Furthermore, in order to control drug activity, tuning the time scale of the reverse thermal isomerization step toward the thermodynamically stable form is also essential. The goal is to develop a photoswitchable drug that is photochemically switched to a more active but less stable isomer upon administration. In the body, it will be effective for a time period defined by the half-life before switching to the less-active but thermodynamically most stable isomer.

While the emblematic azobenzene scaffold possesses some advantageous properties, such as a high extinction coefficient, photostability during many switching cycles, and high efficiency, its use as a drug remains unsettled as it absorbs in the ultraviolet and fails to switch completely from the most stable *E*-isomer to the metastable *Z*-isomer.^12^ Heteroaryl azoswitches are emerging as promising alternatives because of their bistability, i.e., they possess isomers with well separated absorption bands that allow selective control of the photoswitching in both directions.^13,14^ Thus, a number of azoheteroaryls derivatives have been synthesized with thermal *Z*/*E* isomerization rates that correspond to half-lives spanning from minutes^15,16^ up to years.^13,14^

Given its relevance, many efforts have been invested in understanding the mechanism underlying the thermal *Z*/*E* isomerization of azoswitches.^17−21^ Particularly in azobenzene, there has been a controversial discussion about the performance of Eyring transition state theory to calculate activation entropies and thus kinetic rates.^22^ Regardless of the method employed to calculate activation free energies, obtained activation entropies were not in agreement with the experiment.^22^ This puzzle was finally resolved showing that the mechanism of isomerization requires the involvement of triplet states^23,24^—otherwise neglected. The participation of triplet states in the thermal isomerization of azobenzene was actually proposed almost 20 years ago^18,25^ but it has been largely overlooked while studying many azobenzene derivatives, with few recent exceptions.^19−21,23,24^

The goal of the present paper is to investigate the mechanism underlying the thermal *Z*/*E* isomerization of the arylazopyrazole compound phenylazo-1,3,5-trimethylpyrazole (PATP, see Figure 1) a promising photoswitch for photopharmacology.

**Figure 1:**
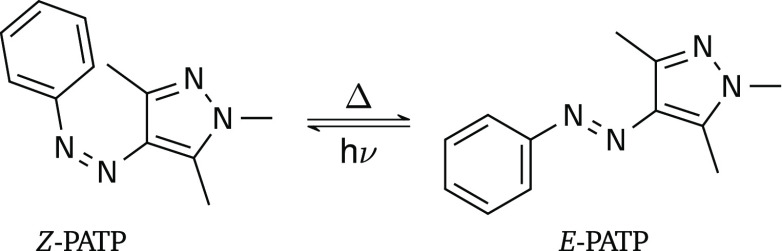
Schematic depiction of the thermal *Z*/*E* isomerization of phenylazo-1,3,5-trimethylpyrazole (PATP).

Based on current literature about azobenzene,^18,23,24^ the thermal isomerization of PATP should be also discussed in terms of inversion and rotation mechanisms involving singlet and triplet electronic states. Figure 2a shows plausible transition states (TSs) associated with the in-plane inversion of either the pyrazole or aryl moieties around their neighboring azo nitrogen (TS_iPy_ with NNC angle α′ ≈ 180° and TS_iAr_ with CNN angle α ≈ 180°) and the out-of-plane rotation around the azo-bond (TS_r_ with CNNC dihedral angle *d* ≈ 90°), respectively. Traditionally, such movements were assumed to exclusively follow the potential energy surface of the singlet electronic ground state S_0_.^12,26−28^ This is true for the inversion mechanisms, resulting in the potential energy profiles schematically shown in Figure 2b. However, along the rotational coordinate it is possible that intersystem crossing from S_0_ to T_1_ and back to S_0_ takes place, giving rise to the coupled potential energy curves displayed in Figure 2c—as proposed for azobenzene.^18,23,24^ In this case, two pathways can be conceived: one that occurs fully in the S_0_ state (red line in Figure 2c and labeled Path_r_) and another that involves both the S_0_ and the T_1_ states (blue line, Path_rT1_). The latter pathway implies spin–orbit coupling and nonadiabatic effects coupling two electronic states—a rather atypical situation for chemical reactions that occur upon thermal activation from the electronic ground state.

**Figure 2:**
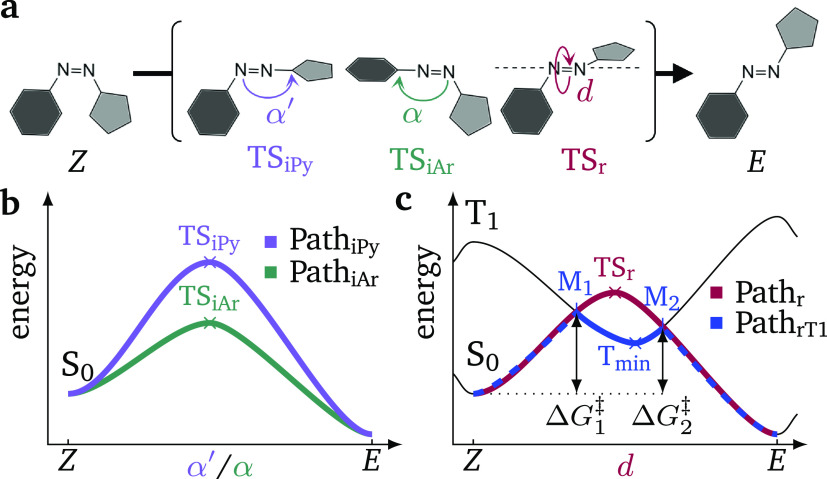
(a) Schematic structures of the inversion and rotation transition states (TSs) of the thermal *Z*/*E* isomerization of PATP. The in-plane angles α/α′ of the inversions and the dihedral angle *d* of the rotation are highlighted. (b–c) The four proposed thermal isomerization mechanisms in azobenzene derivatives: Path_iPy_ (purple) and Path_iAr_ (green) (b), as well as, Path_r_ (red) and Path_rT1_ (blue) (c). Transition states, singlet–triplet minimum energy crossing points M_1_/M_2_, and the minimum of T_1_ (T_min_), as well as activation energy barriers Δ*G*_1_^‡^/Δ*G*_2_^‡^ of the Path_rT1_ are indicated.

Note that, while for the Path_r_ the barrier controlling the thermal isomerization is the transition state TS_r_, in the Path_rT1_, the reaction is governed by the activation barrier to the minimum energy crossings points (MECPs) M_1_ and M_2_ between the S_0_ and T_1_ potentials (Δ*G*_1_^‡^/Δ*G*_2_^‡^ in Figure 2c).

Previous studies on heteroarylazoswitches have either neglected the pathway associated with TS_r_ regardless of spin,^15,16,29^ or considered rotation but neglected triplet states,^26,28,30−32^ or considered only the T_1_ minimum (not the MECPs) to calculate the barrier,^20^ or just suggested the triplet mechanism qualitatively.^19,21^ By contrast, the most recent work^23,24^ on azobenzene argued that the thermal isomerization proceeds through the rotational triplet mechanism, Path_rT1_. In this work, we want to investigate all four thermal isomerization pathways on the same footing in order to clarify whether the triplet pathway is also the dominant mechanism in PATP, as proposed for azobenzene.

To this aim, we employ a protocol based on density functional theory (DFT), backed up by multiconfigurational reference calculations (Sections S1 and S2 of the Supporting Information). Following the characterization of the potential-energy surfaces, we compute reaction rates and half-lives of all reaction pathways using the complementary conventional^33,34^ and nonadiabatic^23,35^ transition-state theories (TSTs). We compare the calculated total reaction half-life with the experimental result from which we conclude that in contrast to azobenzene,^23,24^ the half-life in PATP must be controlled by two pathways with similar time scales: the inversion pathway Path_iAr_ and the rotational path via the triplet state, Path_rT1_. Furthermore, we experimentally validate the involvement of triplet states in the thermal isomerization of PATP by showing that the *Z*/*E* backswitching is considerably accelerated in the presence of a triplet-photosensitizer. As the herein presented triplet state mechanism has not been reported before for arylazopyrazoles, it is especially important to show its feasibility and operation at an experimental example.

As a first step in our endeavor, we characterized the four plausible thermal reactions by identifying the critical points relevant to the four pathways: the reactant and product equilibrium structures (*Z* and *E*) in the S_0_, the T_1_ triplet minimum (*T*_min_), the TSs in the S_0_ (TS_iPy_, TS_iAr_, and TS_r_) and the singlet–triplet MECPs (M_1_ and M_2_). All the critical points were optimized at the ωB97X-D/def2-TZVP@SMD(DMSO) level of theory,^36−40^ except the TS_r_, which was optimized using spin-flip time-dependent density functional theory (SF-TDDFT) due to its multiconfigurational character.^24^ Relaxed potential energy scans in the T_1_ connecting the MECPs M_1_ and M_2_ were performed to characterize Path_rT1_, while unrelaxed scans were carried out to connect the *Z*/*E* isomers with the M_1_/M_2_ points in Path_rT1_ as well as with the TS_r_ in Path_r_ (Section S1).

The electronic energies and geometries of the critical points as well as the scans connecting pathways Path_r_ and Path_rT1_ are summarized in Figure 3. The lowest-lying TS in the electronic ground state S_0_ is TS_iAr_ (green horizontal line), predicted at 26.6 kcal mol^–1^. The TS_iPy_ (purple line) and TS_r_ (red line) are above in energy, at 29.5 and 34.1 kcal mol^–1^, respectively. The TS_iAr_ is characterized by a T-shaped geometry, where the aryl and pyrazole rings are perpendicular to each other, while in the TS_iPy_ the geometry is more twisted. The energetic stabilization of the T-shaped *Z*-conformers of azoheteroarene photoswitches has been discussed already in the literature^15^ and we can expect here a similar steric stabilization for the T-shaped TS_iAr_. These results suggest that—within the S_0_—the thermal isomerization occurs preferentially through the two inversion pathways, as considered in previous literature.^15,16,29^ However, the triplet pathway Path_rT1_ lies lower in energy. From the S_0_ surface until MECP M_1_ one requires only 23.8 kcal mol^–1^ and this pathway continues in the T_1_ surface over a minute barrier (0.2 kcal mol^–1^) until recrossing to the S_0_ at the MECP M_2_, located at 22.2 kcal mol^–1^. The energy barriers for crossing points M_1_ and M_2_ are slightly different due to the lack of symmetry in our arylazopyrazole molecule. We note that the T_1_ minimum, *T*_min_, lies at 22.1 kcal mol^–1^. We also find sizable spin–orbit couplings between the S_0_ and T_1_ states (30.5 and 31.6 cm^–1^) at both crossing points (M_1_ and M_2_, respectively), indicating that Path_rT1_ via intersystem crossing is competitive with the ground-state-only isomerization pathways.

**Figure 3:**
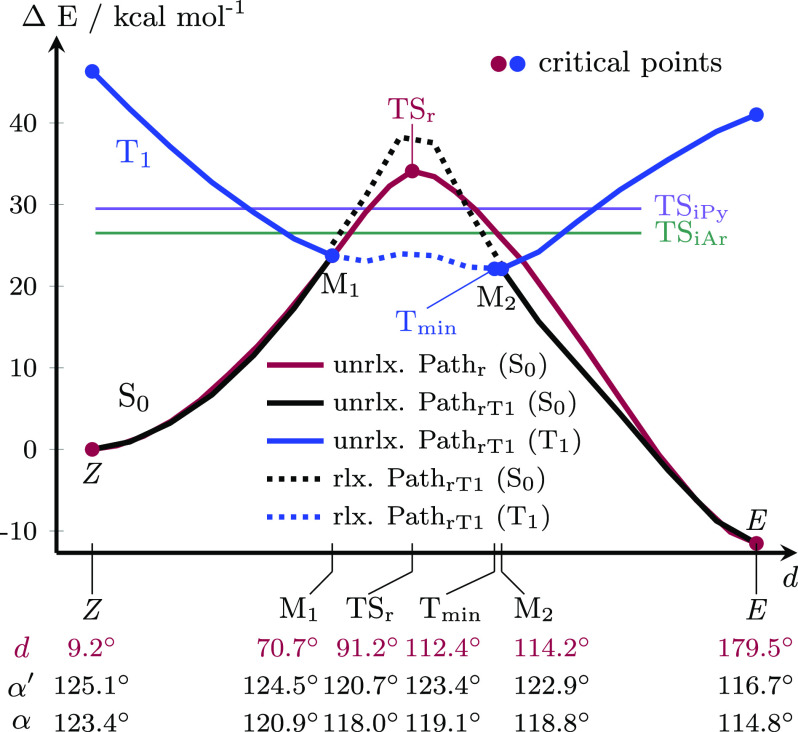
Unrelaxed scan (solid line) along Path_r_ and combination of unrelaxed (solid line) and relaxed (dotted line) scans along Path_rT1_ pathway. Structures correspond to optimized geometries at critical points (large dots). Energies of TS_iAr_ and TS_iPy_ transition states are indicated by horizontal lines. All energies are in kcal mol^–1^ relative to the *Z*-isomer energy.

In order to quantify the role of the four pathways in the thermal isomerization of PATP, we calculated the rates of the ground-state processes using conventional TST^33,34^ as well as the rate of the triplet pathway Path_rT1_ using nonadiabatic TST (NA-TST)^35,41^ (Section S3). The resulting rates and corresponding half-lives at 23 °C as well as the calculated Gibbs free energies Δ*G*^‡^ are collected in Table 1 (Section S4). Surprisingly, the fastest reaction is given by the pathway involving inversion of the phenyl substituent Path_iAr_ with a half-life of 1.3 days—recall that this was the mechanism involving the lowest-lying TS in the electronic ground state S_0_. The triplet pathway Path_rT1_ occurs on a similar time scale, with a half-life of 3.9 days. The other pathways, Path_iPy_ and Path_r_, take place on considerably slower time scales (years). We then also calculated an overall reaction rate as the sum of the rates for each of the independent parallel paths, and this amounts to 0.9 days. Given the accuracy of the calculations, we conclude that the overall half-life is the result of the two pathways, 75% Path_iAr_ and 24% Path_rT1_, with less than 1% involvement of Path_iPy_ and Path_r_. We see similar energetic results for our reference multiconfigurational calculations (Section S2) and are therefore certain that PATP will isomerize through a combination of Path_iAr_ and Path_rT1_.

**Table 1:** Barrier Heights Δ*G*^‡^,^a^ Calculated Reaction Rates *k*(*T*), and Half-Lives τ_1/2_ at the ωB97X-D/def2-TZVP@SMD(DMSO) Level of Theory for the Four Individual Pathways

	Δ*G*^‡^			
	(eV)	(kcal mol^–1^)	*k*(*T*) (s^–1^)	τ_1/2_
Path_iPy_	1.21	27.8	1.8e-08	1.2 a
Path_iAr_	1.06	24.4	6.4e-06	1.3 d
Path_r_	1.41	32.4	7.1e-12	3,097 a
Path_rT1_^b^	0.99	22.8	2.1e-06	3.9 d
	0.92	21.1		
Sum^c^			8.5e-06	0.9 d
Experiment			7.6e-07	10.5 d

a All energies are given relative to Δ*G*(*Z*)= −684.4710 E_h_.

b For Path_rT1_, Δ*G*_1,2_^‡^ of M_1_ and M_2_ as well as *k*_NA-TST_(*T*) are given.

c Sum denotes the overall reaction rate as sum of the four individual rates.

In comparison, our experiments measure a half-life of 10.5 days. This value was determined by tracking the thermal *Z*/*E* isomerization of PATP via regular measurements of the compound’s absorption spectrum over 15 h and extrapolation of that data as well as establishing and extrapolating a respective Eyring plot (Section S5). As the calculations represent an idealized scenario in which all of the reactant molecules undergo the isomerization reaction without experimental limitations, we expect to obtain an upper limit with the theoretical value. Also, given that small errors in the calculation of the electronic energies of the reaction barriers translate into large differences in reaction rates (Section S4), we consider our results to be in reasonably good agreement with the experiment.

As we cannot push the limit of the calculations to discern the role of triplet states in the thermal *Z*/*E* isomerization mechanism of PATP further, we turn to perform photoswitching experiments in the presence of a photosensitizer that can prove the involvement of the triplet state. To this aim, we use methylene blue (MB), which is a photosensitizer with a singlet emission wavelength of 688 nm and a triplet emission wavelength of 867 nm (Section S5).^42−44^ The triplet emission energy of MB is slightly lower than the energy gap between the S_0_ and T_1_ states of the *Z*-isomer, which guarantees that only Path_rT1_ is activated, not any other pathways that involve higher energy states. Despite the slightly lower energy gap of MB in comparison to the S_0_–T_1_ gap of *Z*-PATP, the excitation should be able to induce energy transfer between the triplet state of MB and the triplet state of PATP,^45^ thereby accelerating the *Z*/*E* backswitch, if the triplet pathway Path_rT1_ is operable. Figure 4a sketches the MB activated (photosensitized) isomerization mechanism via intersystem crossing versus the thermal (desensitized) isomerization Path_rT1_. The missing triplet emission energy of MB can be overcome by PATP’s intrinsic vibrational energy and the fact that the energy gap is decreasing on the way to the thermal (desensitized) Path_rT1_.

**Figure 4:**
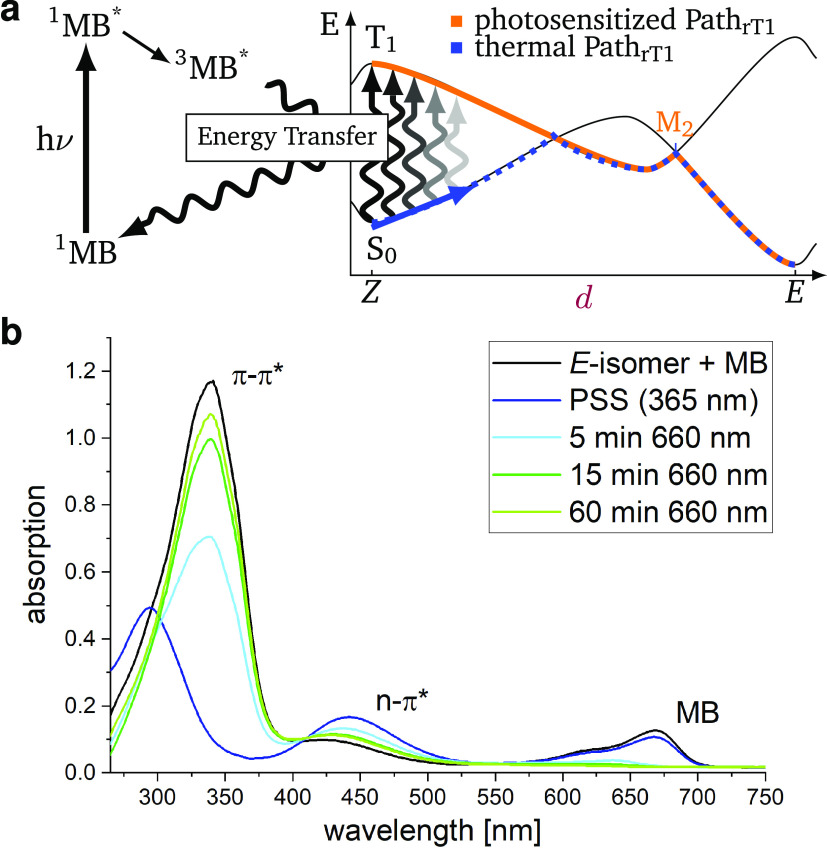
(a) Schematic depiction of the methylene blue (MB) activated thermal *Z*/*E* isomerization (photosensitized Path_rT1_) in comparison to the ’normal’ thermal Path_rT1_. (b) UV–vis spectra of 50 μM PATP in dry DMSO with 10 mol % MB (black line), with π–π* and n−π* transition bands of PATP at 350 and 445 nm, and the MB absorption band at 670 nm.^46^ Spectrum of the photostationary state (PSS, dark blue line) after 365 nm irradiation and time-resolved spectra after 660 nm irradiation of the PSS.

The resulting experimental absorption spectra can be monitored in Figure 4b. Starting from a 10:1 mixture of PATP and MB in DMSO (black line), continuous irradiation at 365 nm reaches a photostationary state (PSS) in the *E*/*Z* photoswitching with high (99%, Section S5.2) *Z*-isomer content (blue line). Irradiation at 660 nm then initiates the photosensitized reaction (light blue, dark green, and light green lines) that leads to a nearly complete *Z*/*E* thermal isomerization and thus to the *E*-isomer. The latter is appreciated by the similarity of the 60 min signal and the initial signal, only missing the MB band. With a higher MB content, even faster *Z*/*E* isomerization can be observed (Section S5). Under these experimental conditions, it is not possible to determine a distinct half-life for the *Z*-isomer, as the *Z*/*E* isomerization seems to quickly slow over time. This problem is likely attributed to photobleaching^47^ and self-aggregation^48^ of the MB at high concentrations, thereby changing efficiency of the energy transfer, as well as oxygen presence in the solution, which can quench triplet states.^49^ Experiments under oxygen-depleted conditions confirmed the last assumption, as there *Z*/*E* isomerization took place even faster (Section S5.3). Photoreduction of MB by the substrate’s pyrazole moiety was considered to also contribute to the observed effect of decreasing MB, but arylamines are known not to cause permanent bleaching.^50^ Nevertheless, the MB-activated species underwent thermal *Z*/*E* isomerization much faster in the range of 1 h compared to the half-life of 10.5 days observed in the absence of MB, confirming the role of triplet states in the thermal isomerization of PATP.

In conclusion, we investigated the thermal *Z*/*E* isomerization of an azoheteroaryl derivative in a combined computational and experimental study. Using a DFT-based protocol backed up by reference multiconfigurational calculations, we examined four possible isomerization pathways, in the singlet and triplet manifolds. Based on reaction rates calculated with conventional and nonadiabatic TST and photosensitization experiments, we find that the thermal *Z*/*E* isomerization of PATP is governed by two pathways at room temperature. One is a conventional ground-state reaction occurring via inversion of the aryl group (Path_iAr_). The other is a nonadiabatic process involving intersystem crossing to the lowest-lying triplet state and back to the ground state, accompanied by torsional motion around the azo bond (Path_rT1_). We thereby show that the reaction rate is not only governed by the smallest activation energy (which is obtained within the triplet-assisted rotational path) but also by the size of the spin–orbit coupling, which in this case slows the rotational mechanism in the triplet state down in favor of the one inversion in the electronically ground state. This conclusion is different from the parent azobenzene, where the rotation mechanism via triplet states has been proposed to be predominant.^23,24^ Whether this is the case for other azobenzene derivatives and other arylazopyrazole photoswitches is an open question. Work along these lines is in progress. In any case, this study clearly highlights the importance of obtaining a full mechanistic picture, including all possible pathways that can contribute to the determination of reaction rates in order to assist the design of photoswitches with tunable thermal half-lives. Further, it is very important to benchmark the theoretical calculations with experimental results in order to identify the obviously varying contributions of the various mechanisms to the overall behavior.

## Supporting Information Available

Computational details of DFT calculations, computational details, results, and assessment of multiconfigurational reference calculations, XYZ coordinates of all optimized compounds, and experimental procedures and characterization data for all new compounds (PDF)
