# Supplementary material for: Singlet and Triplet Pathways Determine the Thermal Z/E Isomerization of an Arylazopyrazole-Based Photoswitch
Source: J Phys Chem Lett. 2023 Sep 29;14(40):8956–61. doi: 10.1021/acs.jpclett.3c01785 (PMC10577781; doi:10.1021/acs.jpclett.3c01785)
Supplement: Supplementary file 1 — jz3c01785_si_001.pdf [file jz3c01785_si_001.pdf]

Supporting Information
Singlet and Triplet Pathways Determine the Thermal Z/E Isomerization of an Arylazopyrazole-Based Photoswitch

Nadja K. Singer,†,‡,§ Katharina Schlögl,¶,§ J. Patrick Zobel,† Marko D. Mihovilovic,*,¶ and Leticia González*,†

†*Institute of Theoretical Chemistry, Faculty of Chemistry, University of Vienna, Währinger Str. 17, 1090 Vienna, Austria*

‡*Vienna Doctoral School in Chemistry (DoSChem), University of Vienna, Währinger Str. 42, 1090 Vienna, Austria*

¶*Institute of Applied Synthetic Chemistry, TU Wien, Getreidemarkt 9, 1060 Vienna, Austria*

§*These authors contributed equally to this work*

E-mail: marko.mihovilovic@tuwien.ac.at; leticia.gonzalez@univie.ac.at

Contents

List of Figures **S4**

List of Tables **S5**

S1 Computational Details **S6**

S1.1 Density Functional Theory S6

S1.2 Additional Single-Reference Single Point Calculations. S9

S1.3 Multiconfigurational Calculations S10

S2 Analysis of the Quantum Chemical Calculations **S16**

S2.1 Energetic Effects on DFT Geometries S16

S2.2 Multiconfigurational effects S19

S3 Transition State Theory **S25**

S3.1 Conventional Transition State Theory S25

S3.2 Non-Adiabatic Transition State Theory S25

S3.3 Wigner Tunneling Transition State Theory S27

S4 Computation of Half-Lives **S28**

S5 Experimental Section **S29**

S5.1 Synthesis of Phenylazo-1,3,5-trimethylpyrazole (PATP) S29

S5.2 Photophysical Characterization of PATP S30

S5.3 Experimental Determination of the $\mathrm{Path}_{\mathrm{rT1}}$ Mechanism S36

S5.4 ^{1}H-NMR and ^{13}C-NMR spectra S44

References **S46**

Appendix **S52**

A1 XYZ Coordinates - DFT S53

A2 XYZ Coordinates - CASSCF S57

A3 XYZ Coordinates - CASPT2 . . . . S60

List of Figures

S1 Conformers . . . S6
S2 DFT geometries . . . S8
S3 Plot of the potential energy scan . . . S9
S4 Active space of Z . . . S12
S5 Active space of M_1 . . . S12
S6 Active space of TS_r . . . S13
S7 Active space of T_{min} . . . S13
S8 Active space of M_2 . . . S14
S9 Active space of E . . . S14
S10 Active space of TS_{iAr} . . . S15
S11 Active space of TS_{iPy} . . . S15
S12 Ground state energies E of critical points (DFT optimized) . . . S17
S13 Optimized geometries . . . S20
S14 Ground state energies E of critical points (CASPT2 optimized) . . . S22
S15 Ground state energies ΔE of critical points optimized with DFT vs CASPT2 S23
S16 Reaction scheme . . . S29
S17 UV/Vis spectra of PATP . . . S32
S18 Experimental kinetic data . . . S33
S19 Experimental kinetic data at 60 °C . . . S33
S20 Experimental kinetic data at 65 °C . . . S34
S21 Experimental kinetic data at 70 °C . . . S34
S22 Eyring plot . . . S35
S23 Experimental setup . . . S39
S24 UV/Vis spectra of PATP with 1mol% MB . . . S39
S25 UV/Vis spectra of PATP with 10mol% MB . . . S40
S26 UV/Vis spectra of PATP with 100mol% MB and pure MB . . . S40

S27 UV/Vis spectra of PATP without MB S41
S28 Experimental kinetic data with 1mol% MB S41
S29 UV/Vis spectra of PATP with 100mol% MB S42
S30 UV/Vis spectra of PATP with 10mol% MB, oxygen-depleted S42
S31 UV/Vis spectra of PATP with 1mol% MB, oxygen-depleted S43
S32 ^{1}H-NMR of **1** S44
S33 ^{13}C-NMR of **1** S44
S34 ^{1}H-NMR of PATP S45
S35 ^{13}C-NMR of PATP S45

List of Tables

S1 Tolerance criteria used for the CASPT2 optimizations S12
S2 Energies ΔE computed on DFT geometries S18
S3 Geometric parameters of DFT, CASSCF, and CASPT2 optimizations S19
S4 RMSD of DFT, CASSCF, and CASPT2 optimizations S20
S5 Energies ΔE computed on CASPT2 geometries S24
S6 Z-content of the PSS at 365 nm and 400 nm S36

S1 Computational Details

S1.1 Density Functional Theory

Optimizations. In order to investigate the different *Z/E* thermal isomerization mechanisms, initial guess geometries for *Z/E*-arylazo-trimethylpyrazole were obtained from conformer searches with *crest 2.11*.[1,2] Then, density functional theory (DFT) optimizations were carried out using the ωB97X-D functional.[3,4] This functional was chosen as it was employed in a previous thermal isomerization study of similar heteroaryl azoswitches[5] and a benchmark paper calculating half-lives of *Z*-azoarenes[6] estimated errors of ca. 2 kcal mol^{-1} when this functional was used in combination with triple-zeta basis sets. Accordingly, we chose the def2-TZVP basis set.[7,8] In order to replicate the experimental conditions, implicit DMSO (dimethylsulfoxide) solvation using the solvation model based on density (SMD)[9] was employed. The ωB97X-D/def2-TZVP@SMD(DMSO)[3,4,7–9] calculations were carried out using the *Gaussian 16* suite.[10]

We focused on the conformer 1 (see Figure S1), where the *N*-methyl group of the pyrazole moiety points away from the aryl moiety in the *Z*-configuration, and neglected the other conformer (conformer 2) that can be obtained by rotation of the pyrazole ring.

Moreover, these types of photoswitches are known to exhibit chirality, particularly helical chirality, which is usually indicated by the (P) or (M) prefixes. We focus our attention on just one of the enantiomers since they possess degenerate energies and the isomerization rates will likewise be the same.

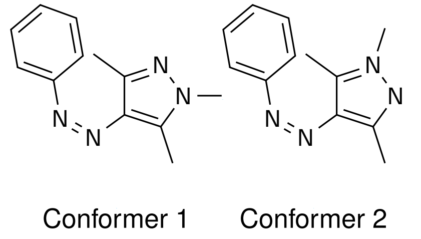

Figure S1: Schematic representation of the 2 possible conformers in the *Z*-configuration.

The same level of theory was used for the optimizations of the inversion transition states

(TSs: TS_{iAr}, TS_{iPy}). The T_1 minimum (T_{min}) was optimized with the Tamm-Dancoff approximation (TDA)[11] based on a restricted Kohn-Sham reference state (S_0). According to Ref. 12, it is important to account for multiconfigurational effects to optimize the rotational TS (TS_r). Therefore, the spin-flip time-dependent DFT (SF-TDDFT) approach was used at the ωB97X-D/def2-TZVP@CPCM(DMSO) level as implemented in *ORCA5.0*.[4,7,8,13–15] The total spin-squared operator, $\hat{S}^2$, for states 1 to 3 of the TS_r was 1.44, 0.78, and 0.12, respectively. States 1 ($\approx T_1$) and 2 ($\approx S_0$) show spin-contamination between pure triplet ($\hat{S}^2=2$) and pure singlet ($\hat{S}^2=0$), which is a common feature in these methods,[16] while state 3 corresponds to the singlet S_1. In our case, these issues arise in the context of using TDDFT open-shell references for the calculation of the TS_r biradical[16] (the rotation in TS_r around the N=N bond breaks the double bond character to form a biradical). States with high spin-contamination ($\hat{S}^2\approx 1$) are undefined states within the SF theory and should be treated carefully.

The minimum energy crossing points (MECPs: M_1, M_2) were optimized at the ωB97X-D/-def2-TZVP@SMD(DMSO) level of theory using the *ORCA 4.2*[13] optimizer and *Gaussian 16*[10] energies by the *SHARC 2.1* code.[17]

All optimized geometries (except for MECPs) were verified to be stationary points by frequency analysis (0 imaginary frequencies for minima, 1 imaginary frequency for TSs). The so obtained geometries (Figure S2) will be referred as DFT geometries in the following and in the main manuscript.

Potential energy scans. Unrelaxed potential energy scans along the *Z*-TS_r-*E*- ($Path_r$) and the *Z*-M_1-T_{min}-M_2-*E*-mechanism ($Path_{rT1}$) were performed. Each scan is an unrelaxed interpolation between the previously DFT-optimized critical-point geometries with 25 single point energy calculations in total for each path at the (TDA-)ωB97X-D/def2-TZVP-@SMD(DMSO)[3,4,7–9] level of theory, as implemented in the *Gaussian 16* suite.[10] The results are shown in Figure 3 of the main manuscript and more extensively in Figure S3.

For the T_1 energy surface the unrelaxed scan shows a small barrier between M_1 and M_2.

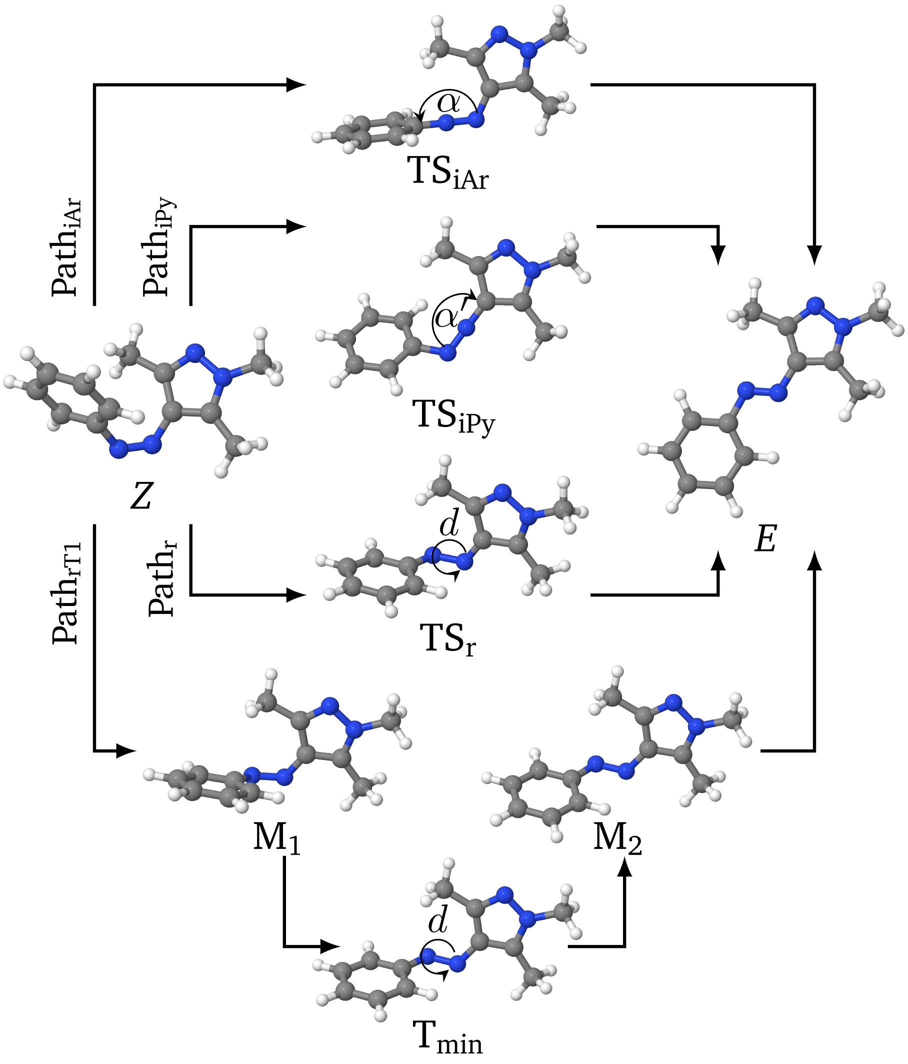

Figure S2: Structures of the DFT(ωB97X-D) optimized critical points along the four Z/E thermal isomerization Paths (Path$_{\mathrm{iAr}}$, Path$_{\mathrm{iPy}}$, Path$_{\mathrm{r}}$, Path$_{\mathrm{rT1}}$). Bonding angles α, α' and azo-torsion angle d are labeled. Cartesian coordinates of all the structures are provided in Appendix A1.

To determine whether this barrier has a physical meaning or is a remnant of the unrelaxed scan we additionally performed a relaxed scan between M$_1$ and M$_2$ in steps of -8.69° (= 6 steps)with the same method and software (see dotted lines in Figures 3 and S3). As the relaxed scan does not show the same small barrier between M$_1$ and M$_2$ we can attribute it to the use of the unrelaxed geometries in the first scan.

Combining more computationally demanding relaxed scans in areas where high accuracy

is needed with less demanding unrelaxed scans in other areas provides an efficient trade-off in accuracy versus computational effort.

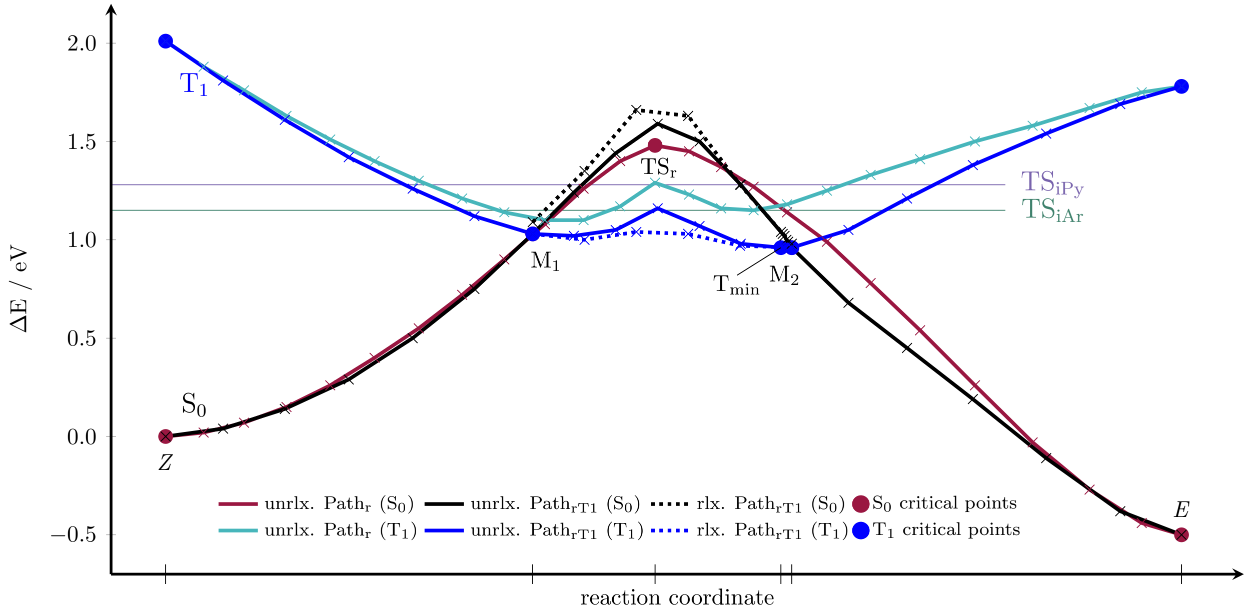

	Z	M_1	TS_r	T_{min}	M_2	E
d	9.2°	70.7°	91.2°	112.4°	114.2°	179.5°
α'	125.1°	124.5°	120.7°	123.4°	122.9°	116.7°
α	123.4°	120.9°	118.0°	119.1°	118.8°	114.8°

Figure S3: Unrelaxed scan (unrlx.) of the Z-TS_r-E-mechanism ($Path_r$, solid red (S_0) and teal (T_1) lines), as well as the Z-M_1-T_{min}-M_2-E-mechanism ($Path_{rT1}$, solid black (S_0) and blue (T_1) lines) plotted against a reaction coordinate. Moreover, a relaxed scan (rlx.) between M_1 and M_2 is shown (dotted black (S_0) and blue (T_1) lines). The critical points (Z, M_1, TS_r, T_{min}, M_2, E) and their S_0 and/or T_1 energies are highlighted by thick dots and their geometrical parameters are given below the plot. For comparison, the barrier heights of the inversion mechanisms (TS_{iAr}, TS_{iPy}) are indicated with gray solid lines. All energies are relative to the Z-isomer ground state energy (-684.6760 E_h).

S1.2 Additional Single-Reference Single Point Calculations.

The effect of other single-reference methods on the energies of the DFT(ωB97X-D) optimized critical points was evaluated using the hybrid functional PBE0[18,19] (the same functional as in the MC-PDFT protocol, vide infra), the hybrid functional PBE0[18,19] with dispersion correction (PBE0-D3),[4,18–20] the 100% Hartree-Fock exchange functional M06HF with dispersion correction (M06HF-D3),[4,21] the double-hybrid functional B2PLYP[22] as well as second-

order Møller-Plesset perturbation theory (MP2).[23] The PBE0,[18,19] PBE0-D3,,[4,18–20] M06HF-D3,[4,21] and B2PLYP-B3BJ[4,20,22,24] calculations were performed using the def2-TZVP basis set[7,8] and implicit SMD(DMSO)[9] solvation with the *Gaussian 16* suite.[10] The MP2 calculations were performed at the RI-MP2/def2-TZVP@CPCM(DMSO)[7,8,25–30] level of theory including the def2-TZVP/C auxiliary basis using *ORCA 5.0.*[13,14]

S1.3 Multiconfigurational Calculations

CASSCF and CASPT2 Single Point Calculations. For comparison, complete-active-space self-consistent-field (CASSCF) calculations were performed at the DFT-optimized critical points. The active space included 14 electrons in 12 orbitals: two π orbitals of the phenyl ring and their π^* counter parts, two π orbitals of the pyrazole ring and their π^* counter parts, the n^+ and n^- orbitals of the azo-group as well as the π and π^* orbitals of the azo-group. The active spaces at the different geometries are shown in Figures S4-S11. We used the state-average approach encompassing 2 singlets or 1 triplet; these two separate calculations will be denoted as (SA(2,1)). The solvent DMSO was included using the PCM implicit solvation model.[31,32] The ANO-RCC-VDZP[33] basis set and the resolution of identity Cholesky decomposition (RICD)[34] approximation were used as implemented in *OpenMOLCAS 21.10.*[35]

The CASSCF energies were corrected with second-order perturbation theory (CASPT2), at the SA(2,1)-CASPT2(14,12)/ANO-RCC-VDZP[33] level of theory. An imaginary shift of 0.2 was used to avoid intruder-state problems.[36]

Furthermore, calculations were performed using IPEA shift values[37] of 0 and 0.25 a.u. Their results will be denoted as CASPT2 and CASPT2$^+$, respectively.

The IPEA shift[37] was introduced into CASPT2[38] in order to correct for a systematic error found in CASPT2 when describing dissociation processes.[39,40] This error was described as a general underestimation of the energy of open-shell electronic states. While this general claim seems to not apply to electronically excited states,[41] using the IPEA shift nevertheless corrects the error in the dissociation in the electronic ground state.[37] Since the description of

the TS_r resembles the electronic structure encountered in ground-state dissociation processes, i.e., a wave function composed mainly of two configurations with similar contributions, it is reasonable to expect the systematic error in non-IPEA-corrected CASPT2 to be present and the energy of TS_r to be underestimated as well. Using the IPEA shift with the recommended shift value of 0.25 a.u.[37] should correct for this underestimation. As it will be shown below, the fact that the IPEA-shifted CASTP2 results agree with the MC-PDFT ones, justifies the use of the IPEA in CASPT2 for this case.

MC-PDFT Single Point Calculations. The CASSCF energies were also corrected using hybrid multiconfiguration pair-density functional theory (MC-PDFT) calculations.[42,43] To this aim, the tPBE0 functional (a translated PBE0[18,19] functional), an ultrafine grid, and the PCM(DMSO) implicit solvation model[31,32] was used, as implemented in *OpenMOLCAS 21.02*.[35]

CASSCF and CASPT2 Optimizations. In order to assess the quality of the DFT geometries, we also performed geometry optimization of the critical points using CASSCF and CASPT2 with the same protocol as described above, except that the geometry optimizations were performed in the gas phase. Subsequent single-point calculations were performed using the same solvent models as described previously. Cartesian coordinates of the resulting structures are listed in Appendix A2 and A3. As a starting guess, the DFT-optimized geometries were employed. These calculations were performed using *OpenMOLCAS 21.02*[35] and the geometry optimization was controlled by the *SHARC 2.1* code[17] using the *ORCA 5.0*[13,14] optimizer.

The CASPT2 optimizations of the Z-isomer and TS_{iPy} did not converge fully using the default convergence criteria in *ORCA*. Instead, the values listed in Table S1 were used.

Table S1: Tolerance criteria used for the CASPT2 optimizations

Structure	Z	TS_{iPy}	all others
Energy change / E_h	-0.0003118000	-0.0000150000	0.0000050000
RMS gradient / E_h $bohr^{-1}$	0.0003592047	0.0006507696	0.0001000000
MAX gradient / E_h $bohr^{-1}$	0.0024268130	0.0061538540	0.0003000000
RMS step / bohr	0.0027859507	0.0124568220	0.0020000000
MAX step / bohr	0.0134615153	0.0358025823	0.0040000000

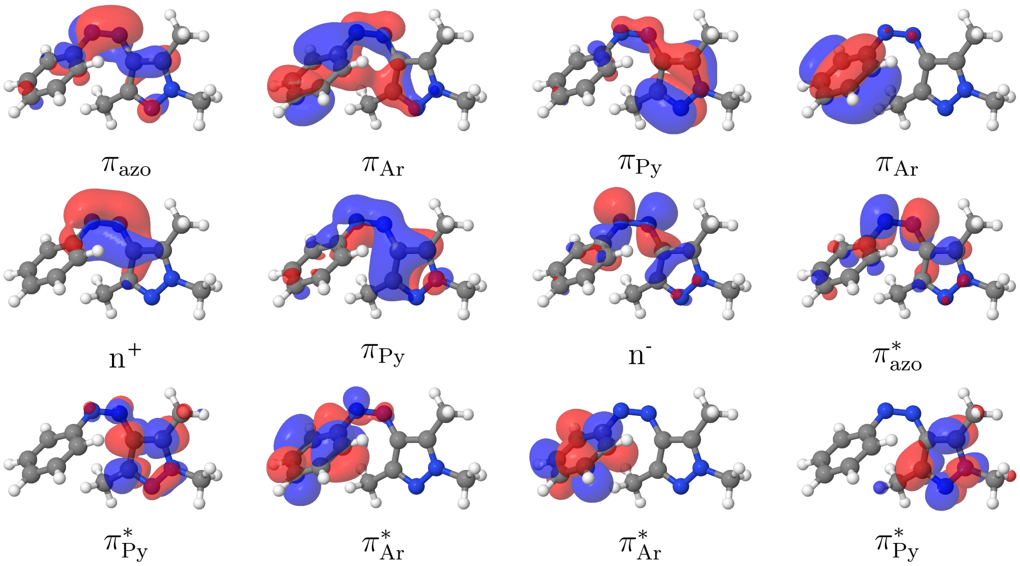

Figure S4: Orbitals included in the active space of the Z conformer.

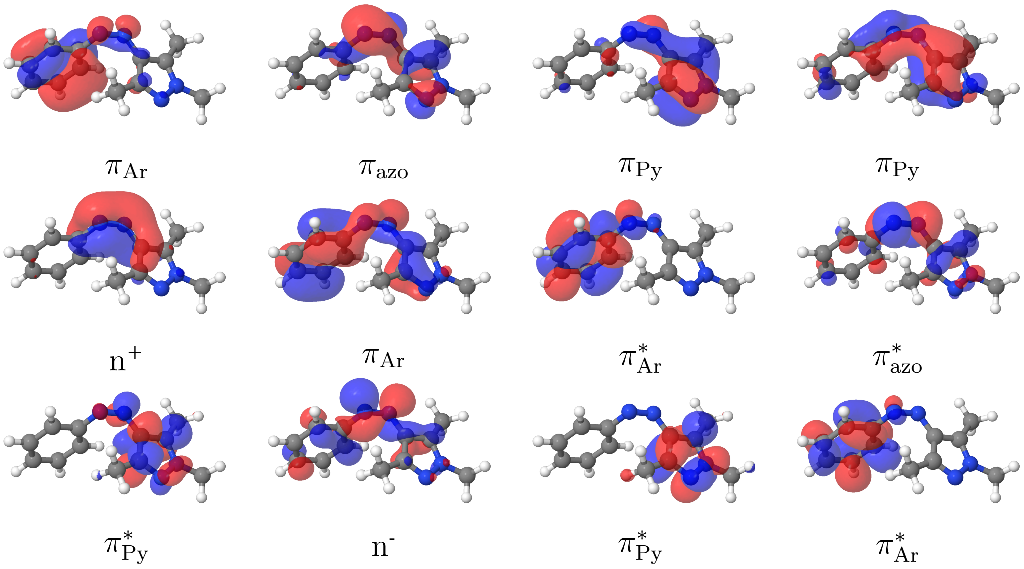

Figure S5: Orbitals included in the active space of the MECP M_1.

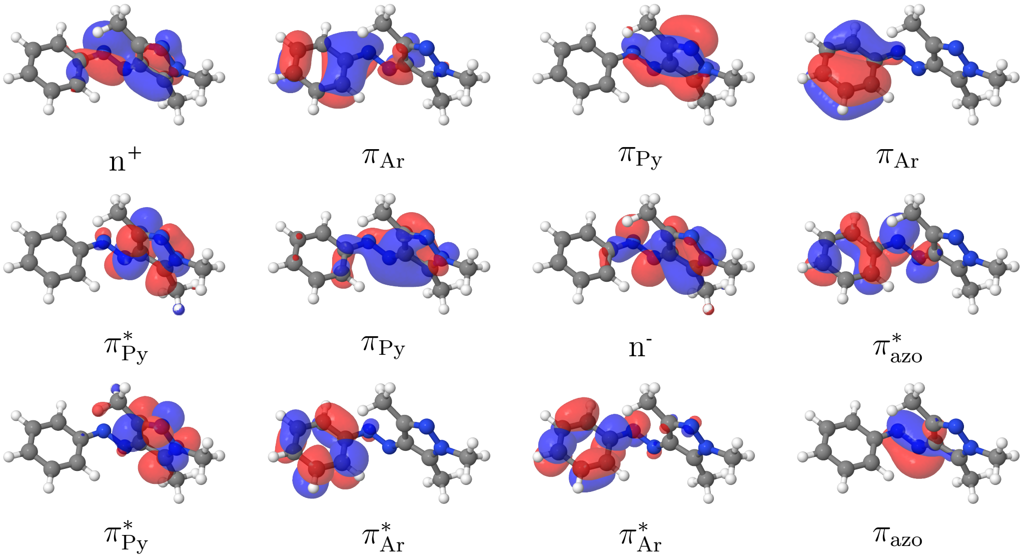

Figure S6: Orbitals included in the active space of TS_r.

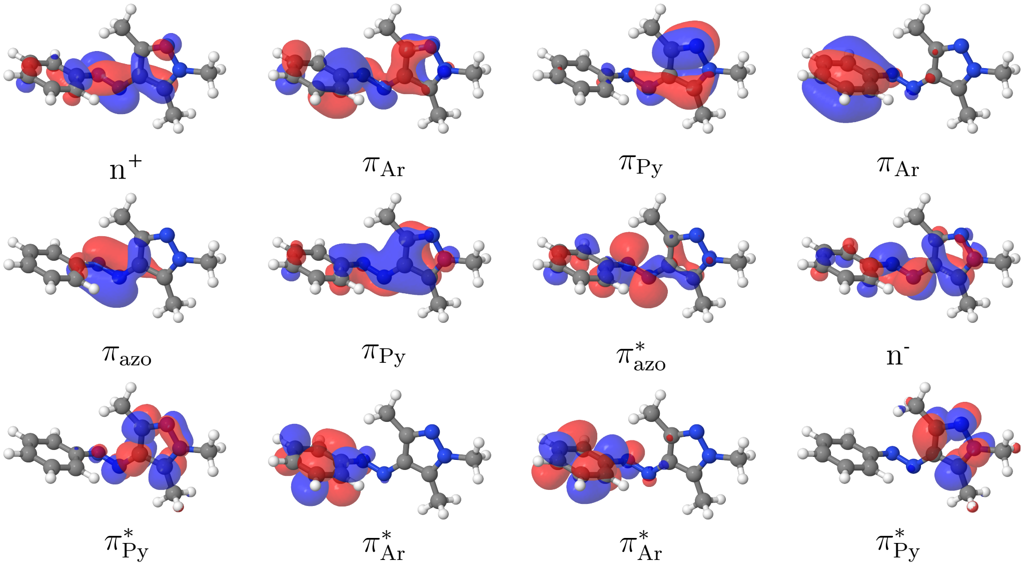

Figure S7: Orbitals included in the active space of T_{min}.

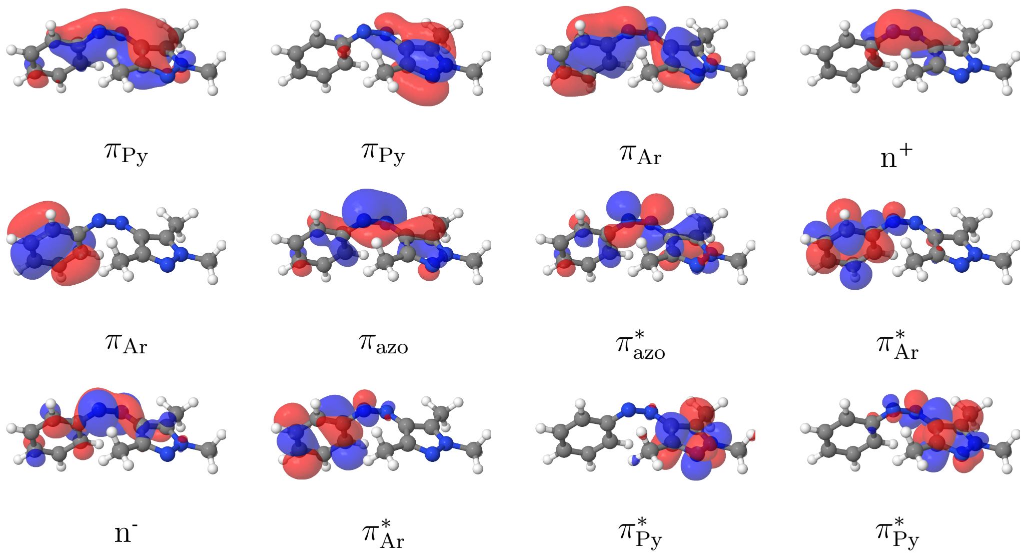

Figure S8: Orbitals included in the active space of the MECP M_2.

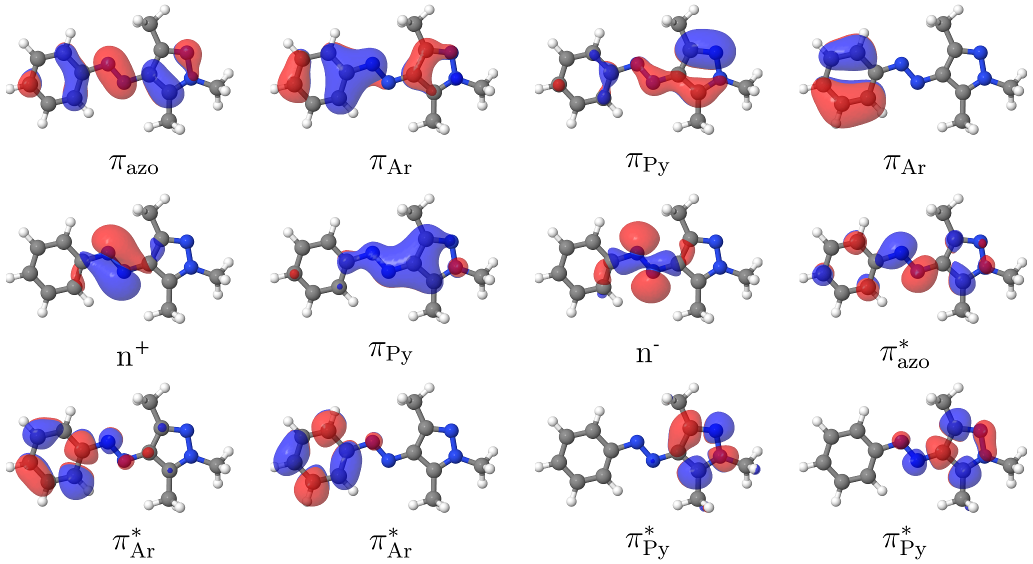

Figure S9: Orbitals included in the active space of the *E* conformer.

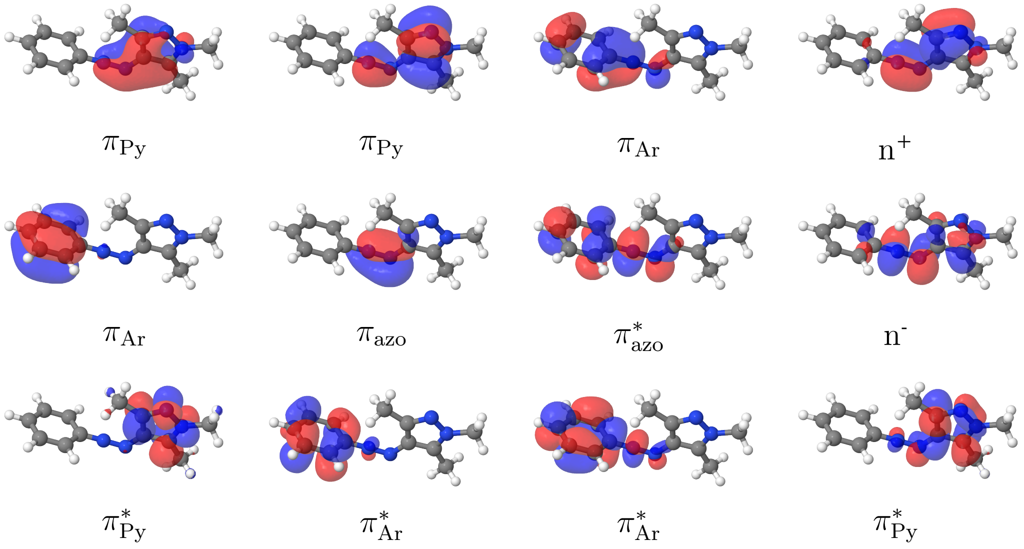

Figure S10: Orbitals included in the active space of $\mathrm{TS_{iAr}}$.

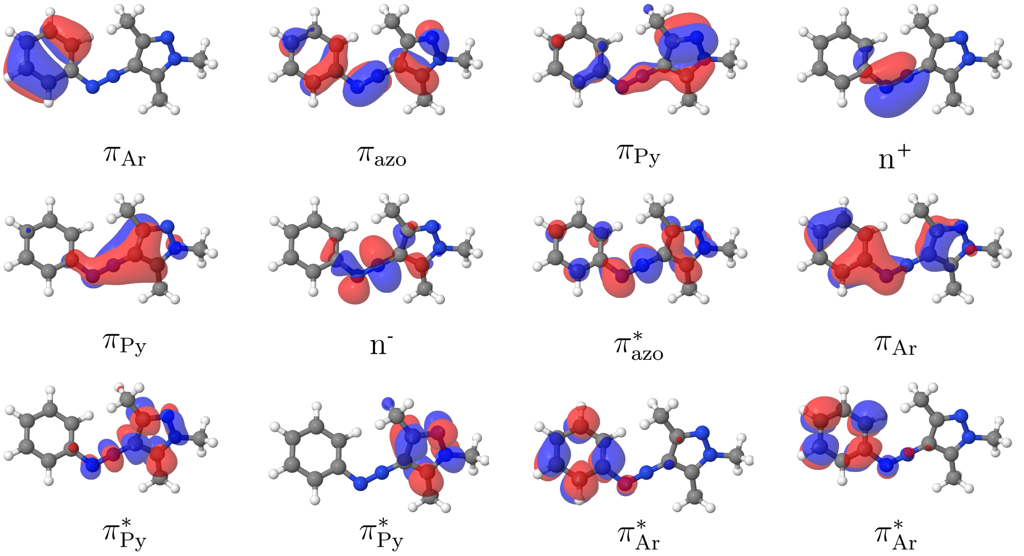

Figure S11: Orbitals included in the active space of $\mathrm{TS_{iPy}}$.

S2 Analysis of the Quantum Chemical Calculations

S2.1 Energetic Effects on DFT Geometries

Here we discuss the energies obtained from the CASPT2/CASSCF and MC-PDFT/CASSCF calculations as well as the single-reference methods, i.e. the DFT calculations using ωB97X-D, PBE0, PBE0-D3, and B2PLYP as well as MP2. In all cases, the energies were obtained as single point calculations on top of the DFT(ωB97X-D) optimized geometries.

The results are collected in Figure S12, showing the relative energies of the important TS_r but also the remaining critical points for comparison. The numerical values are collected in Table S2. At first glance, the relative energy of the TS_r (red line) varies substantially (24-42 kcal mol^{-1}) for the different levels of theory due to the different treatment of static and dynamic correlation. While ωB97X-D, M06HF-D3, PBE0, PBE0-D3, B2PLYP, and MP2 calculations converge within few SCF iterations to single-configurational wave functions, the CASSCF-based methods predict wave functions with two dominant configurations of similar weight for the TS_r. This is expected, confirming the biradical nature of this TS.

Among the latter approaches, CASPT2$^+$ (IPEA shift of 0.25 a.u.) and MC-PDFT predict similar energies for the TS_r (ca. 30.4 kcal mol^{-1}) while CASPT2 (no IPEA shift) underestimates the TS_r with an energy of 24.2 kcal mol^{-1}, likely due to an underestimation of the open-shell ground-state energies, as mentioned in Section S1.3.

Interestingly, despite the different description of the dynamic correlation of TS_r in DFT- and MP2-based calculations, the two DFT variants ωB97X-D and B2PLYP predict a TS_r energy of ca. 34 kcal mol^{-1}, very similar to the CASPT2$^+$ (31.3 kcal mol^{-1}), MC-PDFT (30.4 kcal mol^{-1}), and also the PBE0 (30.7 kcal mol^{-1}) and PBE0-D3 (31.9 kcal mol^{-1}) results. Thus, it appears that the DFT functionals, except for M06HF-D3, are able to profit fortuitously from error cancellation at the TS_r geometry. In contrast, the other single-reference method, MP2, significantly overestimates the TS_r energy (41.7 kcal mol^{-1}) in the same way as M06HF-D3 (42.0 kcal mol^{-1}) does.

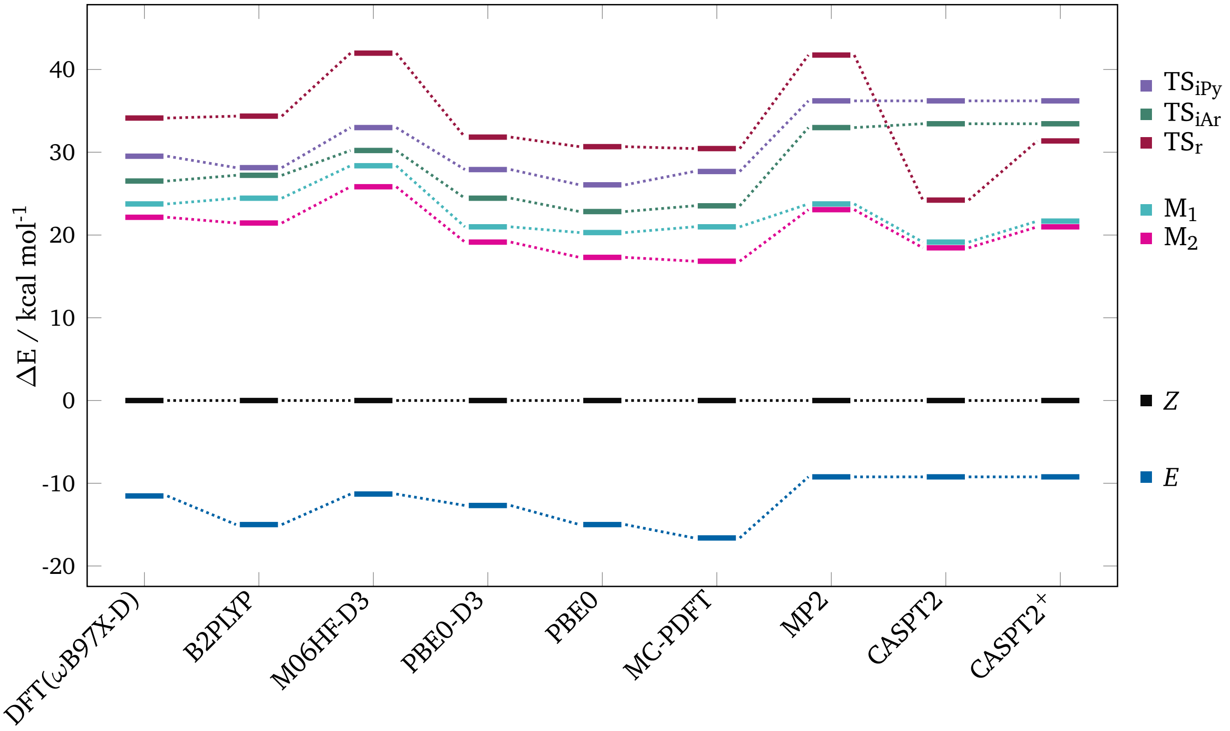

Figure S12: Energies ΔE of critical points relative to the Z-isomer. The structures were optimized with DFT(ωB97X-D) and evaluated at the DFT(ωB97X-D), B2PLYP, M06HF-D3, PBE0-D3, PBE0, MC-PDFT, MP2, CASPT2, and CASPT2$^+$ level of theory (see also Table S2). Dotted lines are only displayed for visual aid.

Regarding the other critical points, it is gratifying to see that all methods predict the crossing points M_1 (turquoise line) and M_2 (pink) below the transition states corresponding to the inversion (TS_{iAr} (green) and TS_{iPy} (purple)), indicating that for PATP the lowest activation energy for the thermal isomerization is provided by the rotational mechanism via triplet states. The DFT calculations , except for M06HF-D3, show similar energies of M_1 and M_2 compared to MC-PDFT with deviations of up to 3.3 and 5.4 kcal mol^{-1}, respectively. The 100% Hartree-Fock exchange functional M06HF-D3 appears to overestimate all energetic barriers of MECPs and TSs. While M_1 and M_2 are similarly described in MP2 and CASPT2 calculations, TS_{iAr} and TS_{iPy} are found at higher energies (6-7 kcal mol^{-1}) when using these perturbation methods, even positioning the inversion TSs above the TS_r for CASPT2$^+$. As both TS_{iAr} and TS_{iPy} should be correctly described by a single configuration, the energetic differences stem from the different treatment of dynamical correlation.

The consistent description of all the DFT-based calculations (ωB97X-D, M06HF-D3, B2PLYP, PBE0, PBE0-D3, MC-PDFT) suggests that the system is better described in the DFT framework compared to the truncated perturbation theory methodologies (MP2, CASPT2), where we experience a complete reordering of the TSs depending on the method.

Focusing on the methods that we consider the most reliable (DFT(ωB97X-D, B2PLYP, PBE0-D3) and MC-PDFT) we see high barriers for $Path_{iPy}$ (27.8-28.2 kcal mol^{-1}) and very high barriers for $Path_r$ (31.9-34.4 kcal mol^{-1}). However, $Path_{iAr}$ shows barriers more accessible at room temperature (24.3-27.3 kcal mol^{-1}). We are therefore certain that a considerable amount of molecules will isomerize through $Path_{iAr}$.

Table S2: Energies ΔE (in kcal mol^{-1}) of the critical points at the DFT(ωB97X-D), M06HF-D3, PBE0, PBE0-D3, B2PLYP, MP2, MC-PDFT, CASPT2, and CASPT2^{+} level of theorys on top of DFT(ωB97X-D) optimized geometries. All energies are given relative to their respective Z ground state energy

structure	state	ωB97X-D	M06HF-D3	PBE0	PBE0-D3	B2PLYP	MP2	MC-PDFT	CASPT2	CASPT2^{+}
Z	S_0	0.0	0.0	0.0	0.0	0.0	0.0	0.0	0.0	0.0
	T_1	46.4	41.7	-	43.8	-	-	49.4	42.5	49.7
	S_1	-	-	-	-	-	-	66.6	63.0	70.1
M_1	S_0	23.8	28.3	20.3	21.0	24.3	23.8	21.0	19.2	21.7
	T_1	23.8	27.1	-	21.0	-	-	24.3	20.0	27.2
	S_1	-	-	-	-	-	-	43.0	41.5	48.5
TS_r	S_0	34.1	42.0	30.7	31.9	34.4	41.7	30.4	24.2	31.3
	T_1	29.7	33.5	-	22.2	-	-	22.3	18.9	26.3
	S_1	-	-	-	-	-	-	36.5	43.2	44.5
T_{min}	S_0	24.1	28.0	19.3	20.9	23.5	25.0	18.6	19.7	22.6
	T_1	22.1	25.8	18.8	-	-	20.8	18.7	25.8	
	S_1	-	-	-	-	-	-	38.7	39.3	46.0
M_2	S_0	22.2	25.8	17.3	19.0	21.4	23.1	16.8	18.4	21.0
	T_1	22.1	26.0	-	18.8	-	-	20.9	18.9	26.0
	S_1	-	-	-	-	-	-	38.8	39.5	46.2
E	S_0	-11.5	-11.3	-14.9	-12.7	-14.9	-9.2	-16.7	-9.3	-9.2
	T_1	41.0	37.9	-	36.5	-	-	38.3	37.1	43.7
	S_1	-	-	-	-	-	-	51.8	52.6	59.2
TS_{iAr}	S_0	26.6	30.2	22.8	24.3	27.3	32.9	23.6	33.5	33.5
	T_1	52.8	49.5	-	46.7	-	-	47.1	46.7	54.5
	S_1	-	-	-	-	-	-	57.4	60.4	67.9
TS_{iPy}	S_0	29.5	32.9	26.2	27.8	28.2	36.1	27.6	36.3	36.2
	T_1	51.9	49.1	-	47.4	-	-	49.9	48.9	56.3
	S_1	-	-	-	-	-	-	61.3	62.3	69.3
ref. energy / E_h		-684.6760	-684.7638	-684.1015	-684.1195	-683.4536	-683.2321	-683.3471	-683.1237	-683.1158

S2.2 Multiconfigurational effects

Geometrical effects. First, we discuss the geometries obtained at the different levels of theory, i.e. the DFT(ωB97X-D), CASSCF and CASPT2. Selected geometrical parameters are reported in Table S3 and the root-mean-square deviations (RMSD) between the atomic coordinates of the differently optimized structures are collected in Table S4. Figure S13 shows the overlap between the three structures. In all cases, it can be seen that the critical points obtained with DFT, CASSCF, and CASPT2 are in good agreement, e.g., with only small differences in the dihedral angle d as well as angles α and α'. Most of the RMSD originates from rotations of the methyl groups. The largest deviation (RMSD=0.2 Å) is obtained for the T_{min} geometry, due to the relatively flat T_1 energy surface between the MECPs (Figure S3); however, these larger geometrical differences do not influence the reaction mechanism.

We therefore conclude that, the geometries involved in the potential energy surfaces of the thermal isomerization of PATP do not seem to be particularly affected by the employed level of theory, or in other words, the DFT geometries are robust and in good agreement with the ones obtained at higher levels of theory.

Table S3: Comparison of geometric parameters of the DFT(ωB97X-D), CASSCF, and CASPT2 optimized geometries

	d / °			α' / °			α / °		
structure	DFT	CASSCF	CASPT2	DFT	CASSCF	CASPT2	DFT	CASSCF	CASPT2
Z	9.15	5.28	9.47	125.07	115.37	123.11	123.37	123.73	121.50
M_1	70.72	74.08	73.76	124.50	122.55	120.12	120.85	120.98	119.72
TS_r	91.24	89.15	90.36	120.74	119.26	116.91	118.01	118.42	116.28
T_{min}	112.35	103.90	96.57	123.39	119.97	120.46	119.07	119.04	119.47
M_2	114.18	109.30	109.48	122.85	119.80	118.90	118.80	119.02	118.36
E	179.54	179.95	179.80	116.70	115.37	113.81	114.81	114.94	113.15
TS_{iAr}	155.86	-	156.94	118.48	-	115.86	179.51	-	180.53
TS_{iPy}	96.95	-	104.97	177.51	-	176.43	116.52	-	115.56

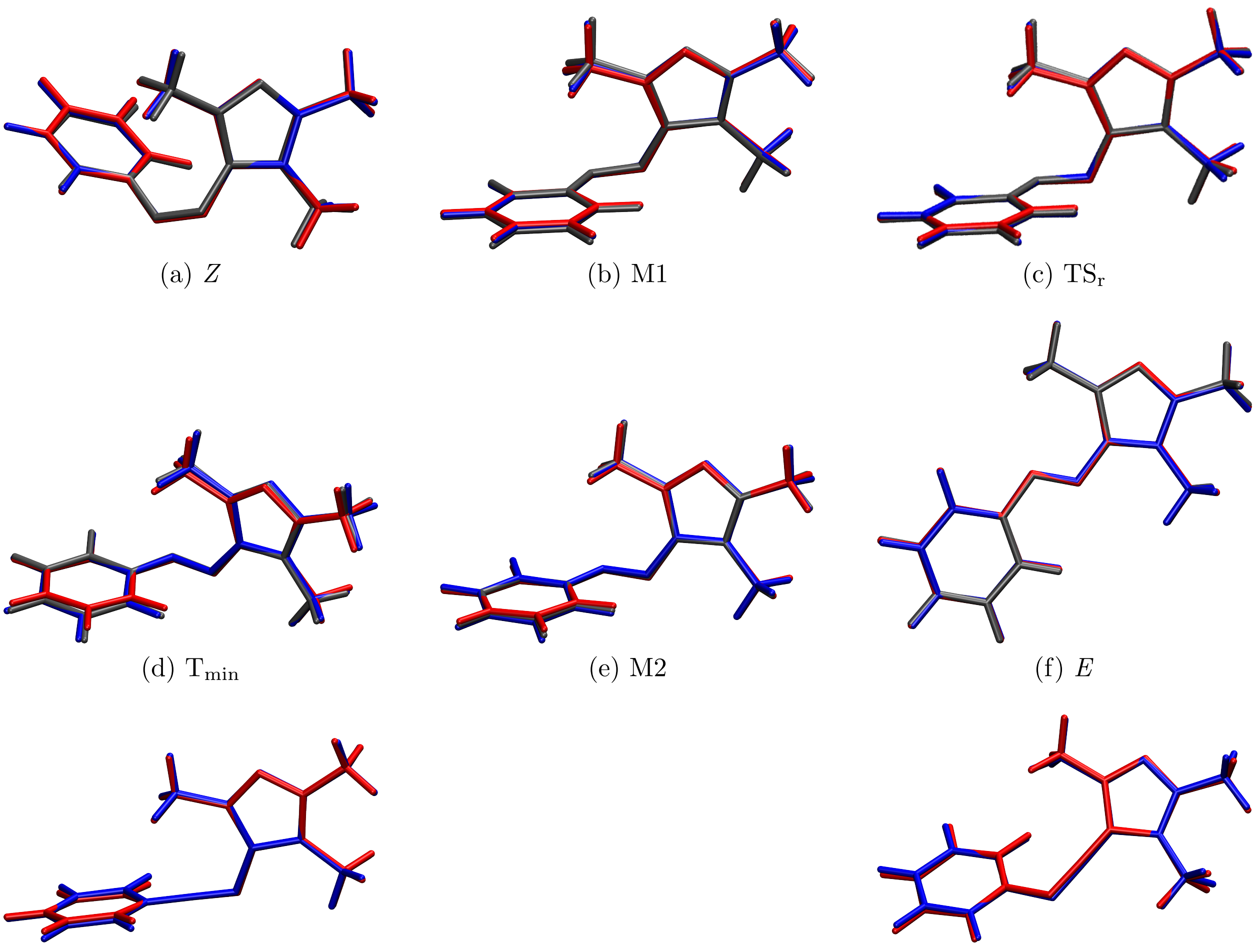

(a) *Z* (b) M1 (c) TS_r

(d) T_{min} (e) M2 (f) *E*

(g) TS_{iAr} (h) TS_{iPy}

Figure S13: Overlay of optimized geometries as indicated obtained from DFT(ωB97X-D) (blue), CASSCF (gray), and CASPT2 (red).

Table S4: Root mean square deviation (RMSD, in Å) of the CASSCF, and CASPT2 optimized geometries relative to the DFT(ωB97X-D) optimized ones

structure	CASSCF	CASPT2
Z	0.157	0.045
M_1	0.076	0.044
TS_r	0.037	0.073
T_{min}	0.110	0.204
M_2	0.064	0.112
E	0.091	0.054
TS_{iAr}	-	0.082
TS_{iPy}	-	0.159

Single Point Energies at the CASPT2-Optimized Geometries. For completeness, we have performed single-point calculations on top of the CASPT2-optimized critical points using our DFT(ωB97X-D), MP2, MC-PDFT, CASPT2, and CASPT2$^+$ protocols. The resulting energies, relative to the Z-isomer, are listed in Table S5 and displayed in Figure S14. Additionally, we compare the DFT(ωB97X-D), MC-PDFT, and CASPT2$^+$ energies against the single-point energies computed at the DFT-optimized geometries (recall Table S2 and Figure S12) in Figure S15. The fact that the DFT and CASPT2 geometries are very similar is translated in very similar energies. Accordingly, when we compare the DFT(ωB97X-D), MC-PDFT, and CASPT2$^+$ energies for geometries optimized with DFT(ωB97X-D) or CASPT2 in Figure S15, we can see that the results within the single point methods are independent of the optimized geometry deviating by only 1.8 kcal mol^{-1} (DFT), 2 kcal mol^{-1} (MC-PDFT), and 0.9 kcal mol^{-1} (CASPT2$^+$). The only exception, with deviations of up to 10 kcal mol^{-1}, is given by the S_0 energy at T_{min} (not shown in Figure S15, see Tables S2 and S5). However, the T_1 energies at the T_{min} only differ by 0.4-2.6 kcal mol^{-1}. The large difference between the S_0 energies at T_{min} is, thus, a consequence of the flat shape of the potential energy surface of the T_1 state (cf. Figure S3). This feature has no influence on the thermal isomerization mechanism, since it does not involve movement in the ground-state S_0 at the T_{min}. Overall, we can hereby confirm our choice of using DFT geometries to predict the different pathways in the thermal isomerization mechanism of PATP.

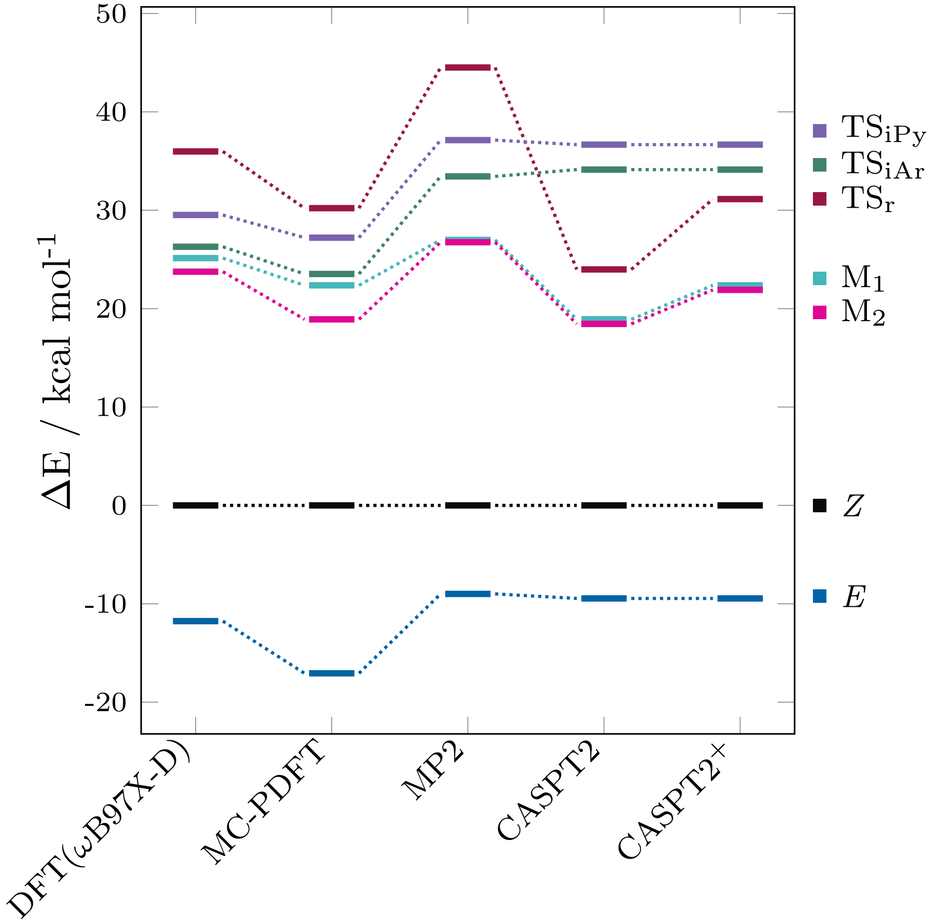

Figure S14: Ground state energies ΔE of critical points. The structures were optimized with CASPT2(no implicit solvation) and evaluated at the DFT(ωB97X-D), MC-PDFT, MP2, CASPT2, and CASPT2$^+$ level of theory. All energies are given relative to their respective Z ground state energy (see Table S5). Dotted lines are only displayed for visual aid.

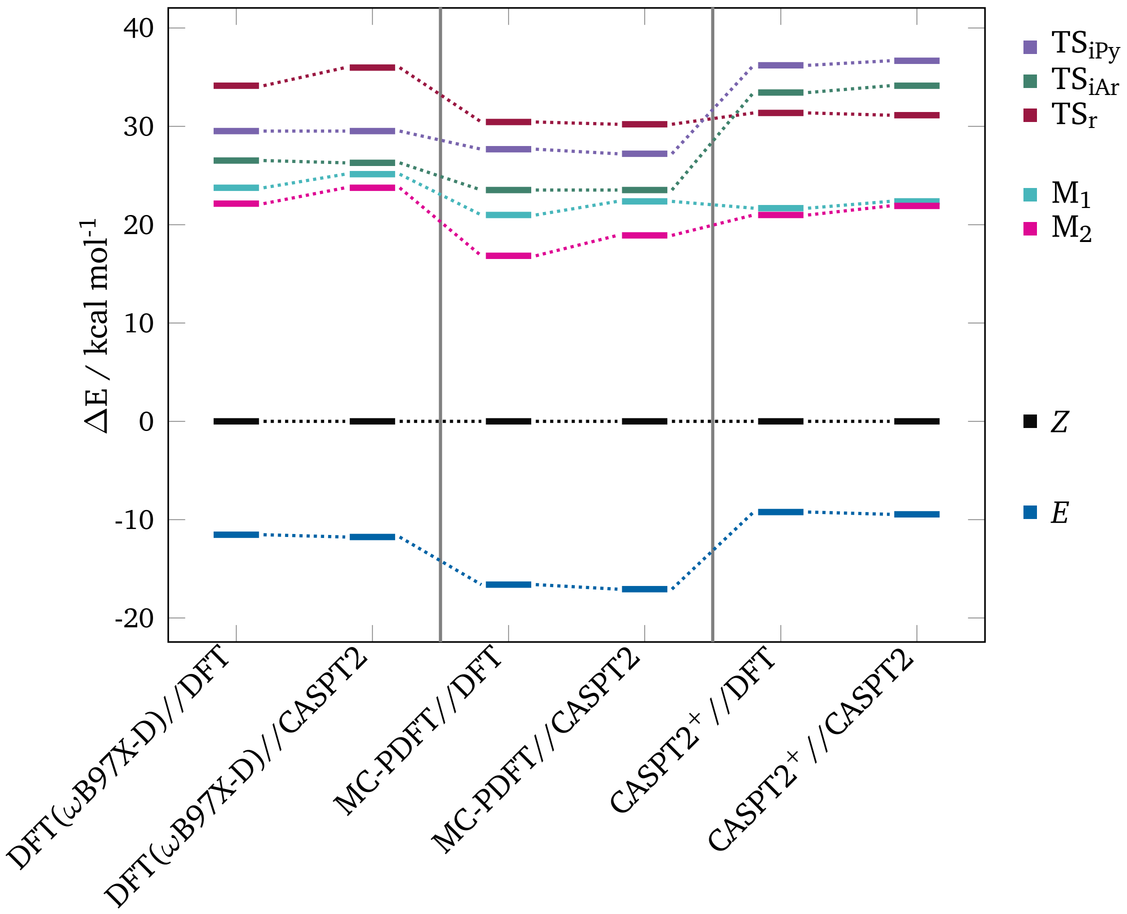

Figure S15: Ground state energies ΔE of critical points. The structures were optimized with DFT(ωB97X-D) or CASPT2(gas phase) and evaluated at the DFT(ωB97X-D), MC-PDFT, and CASPT2$^+$ level of theory (divided by the gray vertical lines). All energies are given relative to their respective Z ground state energy (see Tables S2 and S5). Dotted lines are only displayed for visual aid.

Table S5: Critical point energies E (in kcal mol^{-1}) of structures optimized with CASPT2 and evaluated at the DFT(ωB97X-D), MP2, MC-PDFT, CASPT2, and CASPT2^{+} level of theory. All energies are given relative to their respective *Z* ground state energy

structure	state	ωB97X-D	MP2	MC-PDFT	CASPT2	CASPT2^{+}
Z	S_0	0.0	0.0	0.0	0.0	0.0
	T_1	47.2	-	51.1	44.0	51.3
	S_1	-	-	68.4	64.0	71.3
M_1	S_0	25.1	26.9	22.4	18.9	22.3
	T_1	23.5	-	23.6	19.6	26.8
	S_1	-	-	42.5	44.9	49.8
TS_r	S_0	35.9	44.5	30.2	24.1	31.1
	T_1	30.4	-	22.5	18.8	26.1
	S_1	-	-	39.3	45.6	46.9
T_{min}	S_0	33.4	41.6	28.5	24.9	31.1
	T_1	24.6	-	20.5	18.0	25.3
	S_1	-	-	36.1	43.2	45.2
M_2	S_0	23.7	26.8	18.8	18.4	22.0
	T_1	21.8	-	20.4	18.6	25.8
	S_1	-	-	38.4	42.4	47.3
E	S_0	-11.6	-9.0	-17.0	-9.5	-9.4
	T_1	41.8	-	39.7	38.5	45.2
	S_1	-	-	53.4	54.1	60.7
TS_{iAr}	S_0	26.4	33.5	23.4	34.2	34.2
	T_1	50.8	-	45.8	45.6	53.2
	S_1	-	-	56.6	59.4	66.5
TS_{iPy}	S_0	29.6	37.1	27.1	36.6	36.6
	T_1	48.8	-	47.3	47.1	54.4
	S_1	-	-	59.6	60.9	68.0
ref. energy / E_h		-684.6720	-683.2339	-683.3463	-683.1269	-683.1184

S3 Transition State Theory

The basis of TST is the supposition that a hypersurface exists in phase space that separates the space into a reactant area and a product area. The trajectories that traverse this 'dividing hypersurface' from the reactant to the product space will not cross the hypersurface again (the no-recrossing/dynamical bottleneck assumption).

S3.1 Conventional Transition State Theory

In conventional transition state theory (cTST),[44,45] the surface of division is situated at the saddle point (TS), making the net rate coefficient equivalent to the one-way flux coefficient. According to cTST the rate constant of a thermal *Z/E* isomerization, and therefore the half-life, depends on the Gibbs free energy difference between the stable *Z*-isomer and the TS of the isomerization pathway en route to the *E*-isomer ($\Delta G^{\ddagger}$). We thus set to investigate the *Z/E* isomerization mechanism to obtain the associated rate constant $k_{\mathrm{cTST}}(T)$, assuming a first-order reaction using Eyring equation

$$k_{\mathrm{cTST}}(T) = \frac{k_{\mathrm{B}}T}{h} \exp\left(\frac{-\Delta G^{\ddagger}}{k_{\mathrm{B}}T}\right), \tag{1}$$

with the Boltzmann constant k_{B}, temperature T, and Planck constant h.

S3.2 Non-Adiabatic Transition State Theory

In case of the S_0-T_1-S_0 rotational transition mechanism, we apply the non-adiabatic transition state theory (NA-TST)[46] to obtain the rate constant $k_{\text{NA-TST}}(T)$. NA-TST expands cTST and in our case places two hypersurfaces in the spin-forbidden MECPs instead of the TSs. Moreover, nonrelativistic interactions that cause transitions between states are of importance. For instance, the greater the spin-orbit couplings at the MECPs, the higher the chance of a transition.[46] NA-TST has provided remarkable agreement between experiment in theory in the case of azobenzene.[47] However, similar to cTST, NA-TST is a theory with a

plethora of approximations. Most notably, the no-recrossing (also called dynamical bottleneck) assumption, which states that a reactant reaching the energy barrier to product space can not cross back to the reactant space. We also want to note that NA-TST can be reduced to cTST, by setting the transmission coefficient (γ) to unity and only considering one energy barrier ($\Delta G^{\ddagger}$). Finally the $k_{\text{NA-TST}}(T)$ can be calculated, as

$$k_{\text{NA-TST}}(T) = \frac{k_{\text{B}}T}{h} \frac{\gamma_1 \exp\left(\frac{-\Delta G_1^{\ddagger}}{k_{\text{B}}T}\right) \gamma_2 \exp\left(\frac{-\Delta G_2^{\ddagger}}{k_{\text{B}}T}\right)}{\gamma_1 \exp\left(\frac{-\Delta G_1^{\ddagger}}{k_{\text{B}}T}\right) + \gamma_2 \exp\left(\frac{-\Delta G_2^{\ddagger}}{k_{\text{B}}T}\right)}, \tag{2}$$

where γ_1, γ_2 are the transmission coefficients and $\Delta G_1^{\ddagger}$, $\Delta G_2^{\ddagger}$ the Gibbs free energy difference between the stable Z-isomer and the S_0/T_1 MECPs (M_1, M_2) of the rotational isomerization pathway.[12,47,48]

The transmission coefficient γ is calculated as

$$\gamma = \frac{\pi^{3/2}\alpha}{2\sqrt{\lambda k_{\text{B}}T}} \left[1 + \frac{1}{2} \exp\left(\frac{1}{12\alpha^2(\lambda k_{\text{B}}T)^3}\right)\right], \tag{3}$$

where α is defined as

$$\alpha = \frac{4H_{\text{SO}}^{3/2}}{h/2\pi} \left(\frac{\mu}{F_g|\Delta \mathbf{F}|}\right)^{1/2}, \tag{4}$$

H_{SO} is the spin-orbit coupling, $\Delta \mathbf{F}$ is the singlet-triplet force difference,

$$\lambda = \frac{|\Delta \mathbf{F}|}{2F_g H_{\text{SO}}}, \tag{5}$$

with the geometric mean of the singlet and triplet forces at the crossing

$$F_g = \left|\sum_{n=1}^{N} \sum_{j=1}^{3} (\mathbf{F}_{S_0})_{nj} (\mathbf{F}_{T_1})_{nj}\right|^{1/2}, \tag{6}$$

N is the number of atoms, and the reduced mass μ of the reaction coordinate is given as

$$\mu = \left(\frac{1}{|\Delta \mathbf{F}|^2} \sum_{n=1}^{N} \sum_{j=1}^{3} \Delta \mathbf{F}_{nj}^{\mathbf{2}} m_n^{-1} \right)^{-1}, \tag{7}$$

with m_n being the mass of the nth atom.

Finally, we calculate the total reaction rate of the thermal isomerization as the sum of the rates for each of the independent parallel paths.

$$k_{Z/E}(T) = \sum_{i}^{Paths} k_i(T) \tag{8}$$

The half-lives $\tau_{1/2}$ are calculated from $k_{\text{cTST/NA-TST}}(T)$ by

$$\tau_{1/2} = \frac{\ln(2)}{k(T)}. \tag{9}$$

S3.3 Wigner Tunneling Transition State Theory

With the described version of cTST we neglect the possibility of molecules tunneling through the energy barrier. Because of the relatively large activation energies, a negligible effect on the reactions is expected. However, tunneling might become an important phenomenon for reactions with relatively low energy barriers, since the tunneling probability increases with decreasing barrier height. One possibility to correct cTST[44] reactions rates is to include a one dimensional Wigner tunneling coefficient.[49] We tested the influence of this coefficient using the KiSThelP program[50] (as used by Wang et al.[51]) on the DFT(ωB97X-D) obtained reactions rates for Path_{iAr} and Path_{iPy}. The differences were negligible with reaction rates of 2.2e-8 s^{-1} and 1.8e-8 s^{-1} for Path_{iAr} with and without tunneling, respectively. Similarly, Path_{iPy} has reaction rates of 7.3e-6 s^{-1} and 6.4e-6 s^{-1} with and without tunneling, respectively.

S4 Computation of Half-Lives

In order to compute half-lives within any version of TST, Gibbs free energies are needed. For the DFT(ωB97X-D) level of theory, Gibbs free energy corrections at 23 °C were obtained from frequency calculations on the *Z* isomer and the TSs geometries with the *Gaussian 16* suite. For the activation barriers dictated by the MECPs, frequency calculations for both the S_0 and the T_1 state were performed at the M_1 and M_2 geometries. The difference between the Gibbs free energies obtained from S_0 and T_1 frequency calculations is less than 2 kcal mol^{-1}, so the S_0 Gibbs free energies were used for the calculations of the half-lives. The singlet and triplet forces at the crossing as well as spin-orbit couplings (H_{SO}), needed for NA-TST, for M_1 and M_2 were calculated with *ORCA 4.2*[13] through the *SHARC 2.1* interface (M_1: 30.5 cm^{-1} and M_2: 31.6 cm^{-1}).

We note that computational half-life calculations are chronically difficult, as small variations in the Gibbs free energy barriers can lead to errors of few orders of magnitude in half-lives. For instance, one can compare the Gibbs free energy barrier of 27.8 kcal mol^{-1} of $Path_{iPy}$ with the barrier of 24.4 kcal mol^{-1} of $Path_{iAr}$ (Table 1 in the main manuscript). Even if the barrier difference between $Path_{iAr}$ and $Path_{iPy}$ is only 3.4 kcal mol^{-1}, their respective half-lives are 1.2 years vs. 1.3 days, respectively, illustrating how challenging is this problem for quantum chemistry. While we are aware that the energies obtained by our calculations might not have chemical accuracy to nail down the absolutely correct activation energy and thus the half-live, the fact that we get a consistent energetic order in the transition states with different methods (and anomalies can be explained), makes us confident that our results are qualitatively correct.

S5 Experimental Section

S5.1 Synthesis of Phenylazo-1,3,5-trimethylpyrazole (PATP)

Unless otherwise noted, chemicals were purchased from commercial suppliers and used without further purification. The purity of the reported compounds is > 95% according to NMR. NMR spectra were recorded on a Bruker AC 200 (^{1}H: 200 MHz, ^{13}C: 50 MHz) and Bruker Avance Ultrashield 400 (^{1}H: 400 MHz, ^{13}C: 101 MHz). Chemical shifts are given in parts per million (ppm) and were calibrated with an internal standard of deuterium labeled solvent $CDCl_3$ (^{1}H 7.26 ppm, ^{13}C 77.16 ppm). Thin layer chromatography (TLC) was performed using silica gel 60 aluminum plates containing fluorescent indicator from Merck and detected with UV light at 254 nm. HPLC chromatography was carried out with an Autopurification system of Waters using an ACQUITY QDa Detector in combination with a 2998 Photodiode Array Detector. GC/MS spectra were measured on a Thermo Trace 1300 / ISQ LT (single quadrupole MS (EI)) using a standard capillary column BGB 5 (30 m x 0.25 mm ID). Melting points were determined by a Leica Galen III Kofler and a Büchi Melting Point B-545.

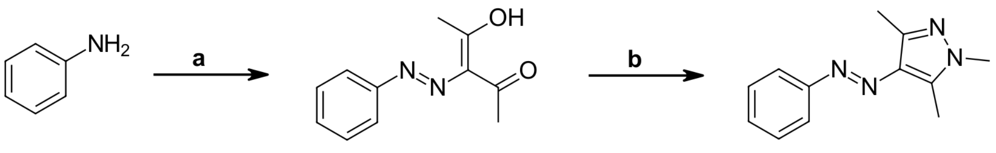

1

2
PATP

Figure S16: Synthetic path to compounds **1** and **2** (=PATP). Conditions: a) AcOH, HCl, $NaNO_2$ (1.2 equiv.), H_2O, -5 °C, 1 h, then acetylacetone (1.3 equiv.), NaOAc (3 equiv.), $EtOH/H_2O$ 2/1, rt, 1 h, 73%, b) methylhydrazine (1 equiv.), EtOH, reflux, 3 h, quantitative.

Synthesis of 4-hydroxy-3-(phenyldiazenyl)pent-3-en-2-one (1)

Compound **1** (see Figure S16) was prepared according to a modified literature procedure.[52] $NaNO_2$ (0.61 g, 8.88 mmol, 1.2 equiv.) was dissolved in H_2O (2.5 mL) and added dropwise to a solution of aniline (0.69 mL, 7.40 mmol, 1 equiv.) in acetic acid (10 mL) and

conc. HCl (1.7 mL) at -5 °C. The resulting mixture was stirred at -5 °C for 1 hour. Then, the solution was added to a suspension of acetylacetone (0.98 mL, 9.62 mmol, 1.3 equiv.) and NaOAc (1.82 g, 22.2 mmol, 3 equiv.) in EtOH (7 mL) and H_2O (4 mL). The reaction mixture was stirred at room temperature for 1 hour. Then, the precipitate was collected by filtration and washed with cold H_2O followed by cold H_2O/EtOH (1/1). After drying *in vacuo*, azo intermediate **1** was obtained as yellow crystals (73%, 1.10 g); mp: 86.0 – 86.5 °C. ^{1}H-NMR (200 MHz, $CDCl_3$): δ = 2.49 (s, 3H), 2.61 (s, 3H), 7.13 – 7.25 (m, 1H), 7.36 – 7.45 (m, 4H), 14.73 (br s, 1H) ppm. ^{13}C-NMR (101 MHz, $CDCl_3$): δ = 26.7, 31.7, 116.3 (2C), 126.0, 129.7 (2C), 133.3, 141.6, 197.1, 198.0 ppm.

Synthesis of phenylazo-1,3,5-trimethyl-pyrazole (2)

Compound **2** (see Figure S16) was prepared according to a modified literature procedure.[52] Compound **1** (1.00 g, 4.90 mmol, 1 equiv.) was dissolved in EtOH (25 mL) and methylhydrazine (0.26 mL, 4.90 mmol, 1 equiv.) was added. The resulting solution was stirred under reflux for 3 hours. Evaporation of the solvent *in vacuo* afforded the desired product **2** as yellow crystals (quant., 1.05 g); mp: 61.0 – 61.5°C. MS (EI, 70 eV): m/z = 215 (10), 214 (76, M+), 137 (100), 109 (86). ^{1}H-NMR (400 MHz, $CDCl_3$): δ = 2.51 (s, 3H), 2.58 (s, 3H), 3.78 (s, 3H), 7.33 – 7.40 (m, 1H), 7.43 – 7.48 (m, 2H), 7.76 – 7.80 (m, 2H) ppm. ^{13}C-NMR (101 MHz, $CDCl_3$): δ = 10.1, 14.0, 36.1, 121.9 (2C), 129.0 (2C), 129.4, 135.3, 138.8, 142.6, 153.7 ppm.

S5.2 Photophysical Characterization of PATP

A UV-1800 UV/Vis spectrophotometer from Shimadzu was used for the measurement of the photophysical properties of PATP. Spectra were recorded in a range from 265 nm to 600 nm at 23 °C for the determination of the photostationary states (PSS) (Figure S17). For irradiation of the samples, OmniCure® LED heads of 365 nm, 385 nm, 400 nm and 460 nm

were used, set to full power (OmniCure® LX400, max. power 320 mW). 50 µM samples in dry DMSO were typically irradiated for 5 seconds from the top to reach the PSS.

After the determination of PATP's PSS, its thermal half-life was measured. Two different methods were used, yieldig very similar half-life times. For an initial, fast half-life time measurement, a 50 µM sample in dry DMSO was preirradiated with 365 nm for 5 seconds. Then, the absorption at 339 nm, the E-isomer's absorption maximum, was regularly measured over the course of 15 hours at 23 °C. This data was linearized according to equation

$$A_{linearzied} = ln(A_{max} - A_t). \tag{10}$$

The linearized absorption data was plotted over time and by applying a linear fit function, rate constant k was gained as slope value (Figure S18). The half-life of 10.5 days was calculated according to equation

$$\tau_{1/2} = \frac{\ln(2)}{k(T)}. \tag{11}$$

To confirm this value, additionally the thermal half-life time of PATP at 60 °C, 65 °C and 70 °C was measured. Again, 50 µM samples in dry DMSO were preirradiated with 365 nm for 5 seconds and the absorption at 339 nm was regularly measured. An exponential fit function was applied to each measurement yielding the rate constant k at the respective temperature as slope value (Figures S19 - S21). This data could then be plotted in an Eyring plot (Figure S22), allowing extrapolation of the half-life time at 23 °C. The thereby obtained value for the half-life time of 10.6 days at 23 °C closely resembled the half-life of 10.5 days observed by the first method. Additionally, the Eyring plot allowed for the calculation of the enthalpic and entropic contribution to the activation energy. The slope value of -11019.4 yielded 91.6 kJ mol^{-1} for the enthalpy of activation $\Delta H^{\ddagger}$ according to the equation

$$slope = \frac{-\Delta H^{\ddagger}}{R}. \tag{12}$$

Respectively, the intercept value of 17.4 led to a calculated entropic contribution $\Delta S^{\ddagger}$ of -52.7 J K^{-1} mol^{-1} according to the equation

$$intercept = ln\frac{\kappa k_B}{h} + \frac{-\Delta S^{\ddagger}}{R}\,, \tag{13}$$

whereby κ was assumed to be 1. At a temperature of 23 °C the entropic contribution equals -15.6 kJ mol^{-1}. The enthalpic and entropic parts of activation thereby added up to a total activation energy of 107.2 kJ mol^{-1} or 25.6 kcal mol^{-1}, being in the range of the theoretical calculations (see Table 1).

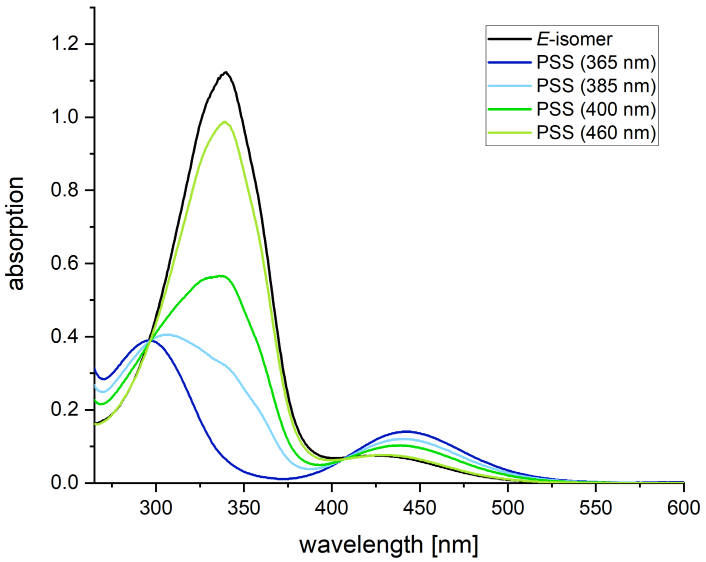

Figure S17: UV/Vis spectra of PATP in the dark (= *E*-isomer, black line) and after reaching its PSSs for several wavelengths.

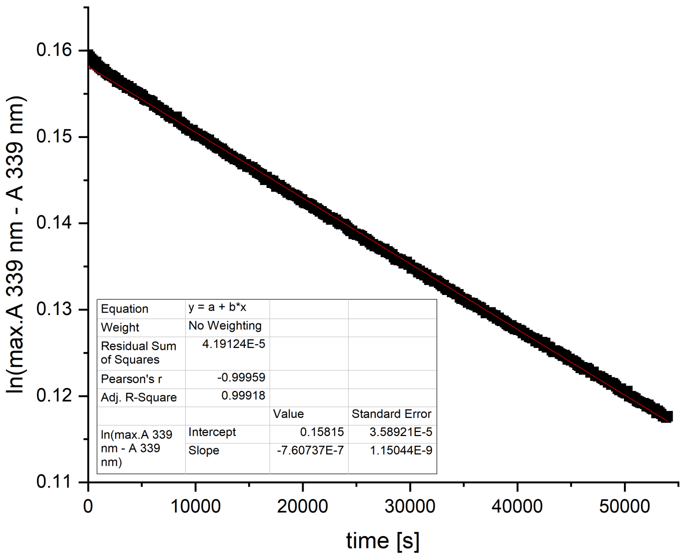

Figure S18: Experimental kinetic data for the thermal *Z/E* isomerization of PATP.

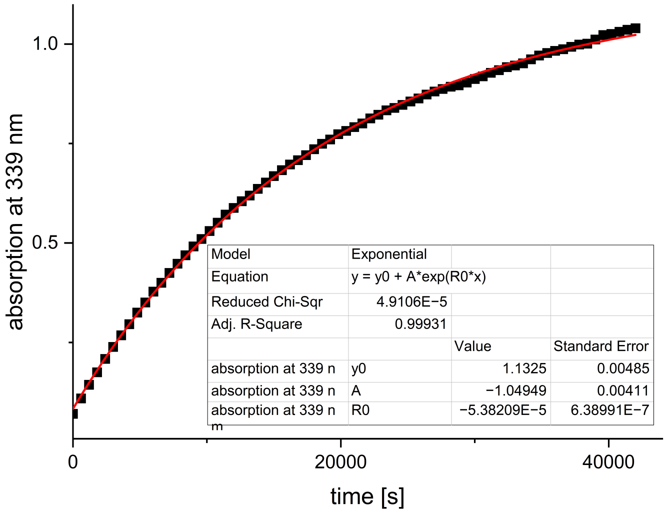

Figure S19: Experimental kinetic data for the thermal *Z/E* isomerization of PATP at 60 °C.

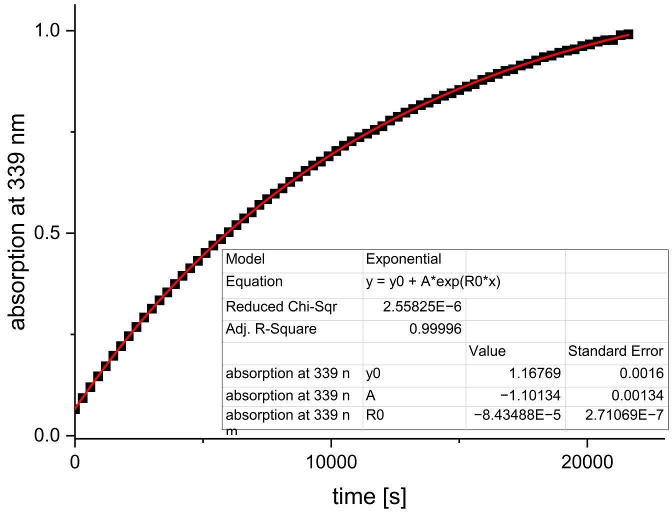

Figure S20: Experimental kinetic data for the thermal Z/E isomerization of PATP at 65 °C.

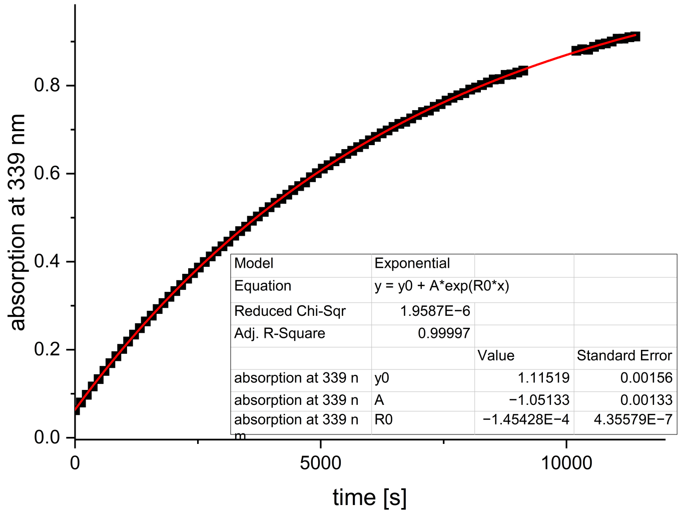

Figure S21: Experimental kinetic data for the thermal Z/E isomerization of PATP at 70 °C. Short interruption during data aquisition around 10000 s visible.

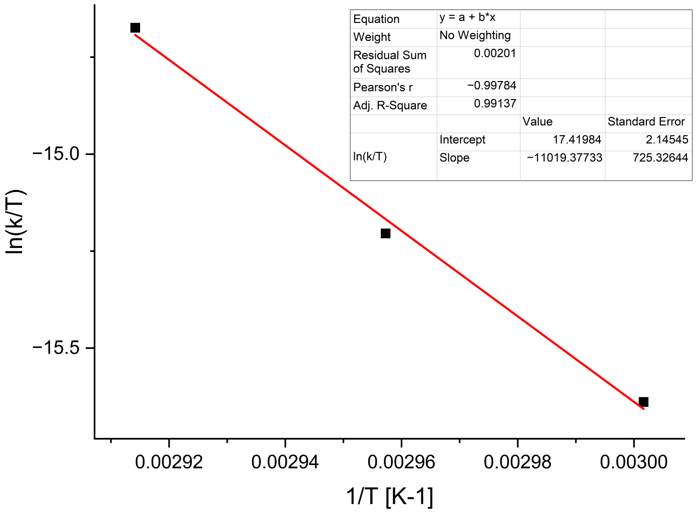

Figure S22: Eyring plot of PATP in DMSO.

Calculation of *Z*-content of PATP's PSS at 365 nm.

The Z-content of PATP was calculated according to a method by Fischer.[53] With this method, one can calculate PSSs of systems A $\rightleftharpoons$ B, in our case E- and Z-isomer, when only A (E-isomer) is known. The experimental data that is necessary for the method by Fischer are absorption spectra of pure species A (equals E-isomer) before irradiation and absorption spectra of PSSs at two different wavelengths. This data was available to us (Figure S17). The method by Fischer assumes that the ratio of the quantum yields ϕ_E/ϕ_Z, ϕ_E being the quantum yield of E to Z isomerization and ϕ_Z being the quantum yield of Z to E isomerization, does not differ at the two chosen wavelengths, which is generally the case. Therefore, the Z-content of PATP could be calculated for all PSSs that experimental spectra had been measured of. As we were especially interested in the Z-content of the PSS at 365 nm, we used the PSS at 365 nm and 400 nm for the calculations (see Table S6). Thereby we could determine a Z-content of 99% in the PSS at 365 nm and a Z-content of 52% in the PSS at 400 nm.

Table S6: *Z*-content of PATP's PSS at 365 nm and 400 nm calculated according to a method by Fischer.[53]

	all E	PSS (365 nm)	PSS (400 nm)	$Z\%_{\text{PSS 365 nm}}$	$Z\%_{\text{PSS 365 nm}}$
A at 365 nm	0.556	0.014	0.267	99%	52%
A at 400 nm	0.068	-	0.054		

S5.3 Experimental Determination of the Path$_{\mathrm{rT1}}$ Mechanism

A UV-1800 UV/Vis spectrophotometer from Shimadzu was used for the measurement of the influence of the Path$_{\mathrm{rT1}}$ on PATP's thermal Z/E isomerization mechanism. Spectra were recorded in a range from 200 nm to 800 nm at 23 °C but are only displayed in the relevant area from 265 nm to 750 nm. For irradiation of the samples OmniCure® LED heads of 365 nm and 460 nm were used, set to full power (OmniCure® LX400, max. power 320 mW). Additionally, a 660 nm red diode laser module RLDH660-70-3, ordered at Roithner Lasertechnik, set to full power (70 mW) was used for irradiation.

Samples of 50 µM PATP in dry DMSO were used for the measurements and photosensitizer methylene blue (MB) was added in either 1, 10 or 100mol% prior to first irradiation.

After sample preparation and initial recording of a dark-state adapted E-isomer UV/Vis spectrum, the samples were photoswitched by irradiation with 365 nm for 15 seconds. A UV/Vis spectrum was recorded of that state, equaling maximum Z-content. Afterwards, the cuvette with the sample was placed underneath the 660 nm laser (3 mm distance between laser and cuvette top) and irradiated (see Figure S23 for experimental setup). After certain time intervals of 660 nm irradiation, intermediary UV/Vis spectra were recorded. Those time intervals were: 5 min, 5 min (total 10 min), 5 min (total 15 min), 15 min (total 30 min), 15 min (total 45 min), 15 min (total 60 min), 60 min (total 120 min). Those intervals were, if necessary, adjusted to the specific sample.

As can be seen in Figure S25, the Z/E isomerization can be significantly enhanced by providing MB as photosensitizer together with constant irradiation with 660 nm. Moreover, we could observe a strong correlation between the content of MB in the sample and the speed of the Z/E isomerization (see Figure S24 to Figure S26). In addition to those experiments, negative controls were measured. Therefore a 50 µM sample of PATP in dry DMSO was prepared and no MB was added, while keeping the irradiation protocol with 660 nm as mentioned above. We observed an accelerated Z/E isomerization similar to the one observed in the sample with 1mol% MB (see Figure S24 and Figure S27). This meant that irradiation with 660 nm led to some increase in the speed of the Z/E isomerization, however not comparable to the effect observed when we applied 10mol% or 100mol% of MB. This also meant, that addition of only 1mol% MB had a neglectable effect on the Z/E isomerization, as the observed faster relaxation for that sample was mostly induced by the 660 nm laser irradiation itself. Additionally, one sample was prepared with 1mol% MB that was not irradiated with 660 nm after initial photoswitching towards the Z-isomer with 365 nm. The half-life of that sample was determined according to the procedure mentioned in Section S5.2 (see Figure S28). The observed half-life was 10.8 days and thereby very close to the determined half-life

without a photosensitizer. As our earlier results had already foretold that 1mol% MB hardly influences the *Z/E* isomerization even with 660 nm irradiaton, we additionally prepared a sample with 100mol% MB that was kept in the dark for 30 minutes after the initial irradiation with 365 nm. Without irradiation with 660 nm, no significant *Z/E* isomerization could be observed in that sample during that time interval (see Figure S28).

Finally, two key experiments were repeated with 50 µM PATP samples in degassed (=oxygen-free), dry DMSO keeping the sample solutions as inert as possible by establishing argon layers. The repeated experiments were the ones with addition of either 1 or 10mol% of MB. Thereby, we sought to eliminate potential quenching of the triplet state by oxygen in the solution. Under oxygen-depleted conditions and addition of 10mol% MB, we observed an even more accelerated *Z/E* isomerization than under ambient conditions (see Figure S30). This showed that the ISC-enhanced *Z/E* isomerization benefits from oxygen-depleted conditions due to less triplet state quenching but is not limited by the presence of ambient oxygen. The oxygen-depleted experiment with 1mol% MB on the other hand showed very similar behavior to the sample with 1mol% MB under ambient conditions (see Figure S31). This might be due to some residual oxygen in the solution or disruptions of the argon layer during the experiments. As the overall concentration of MB in the 1mol% MB experiment equals 0.5 µM, such traces might be enough to still cause photobleaching. The MB concentration dependency of the *Z/E* isomerization acceleration was again highlighted as well as that 660 nm irradiation alone has a significantly lower effect on the *Z/E* isomerization than irradiation together with 10mol% MB.

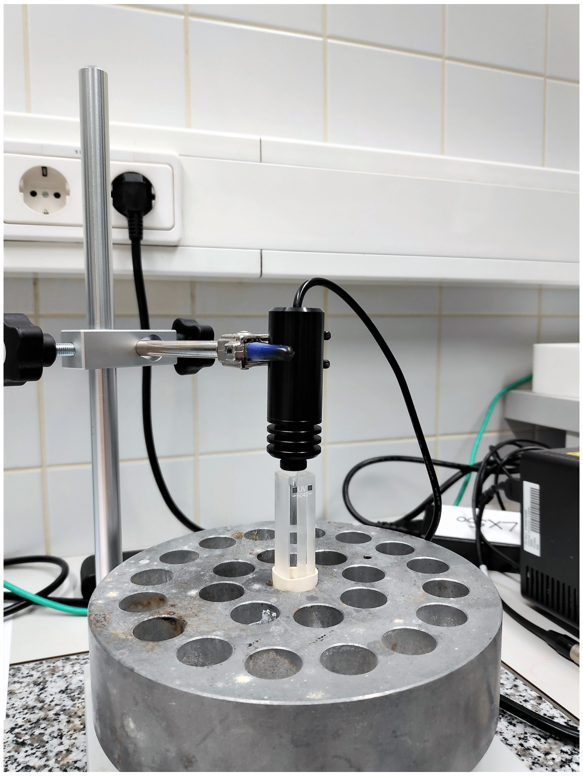

Figure S23: Experimental setup for continuous 660 nm irradiation of the sample solution in the cuvette (here empty).

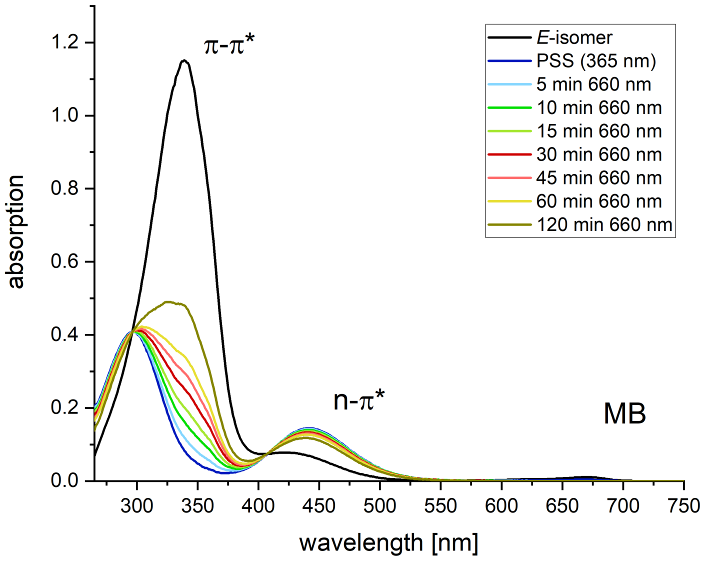

Figure S24: UV/Vis spectra of a 50 µM sample solution of PATP with 1mol% MB after several irradiation steps.

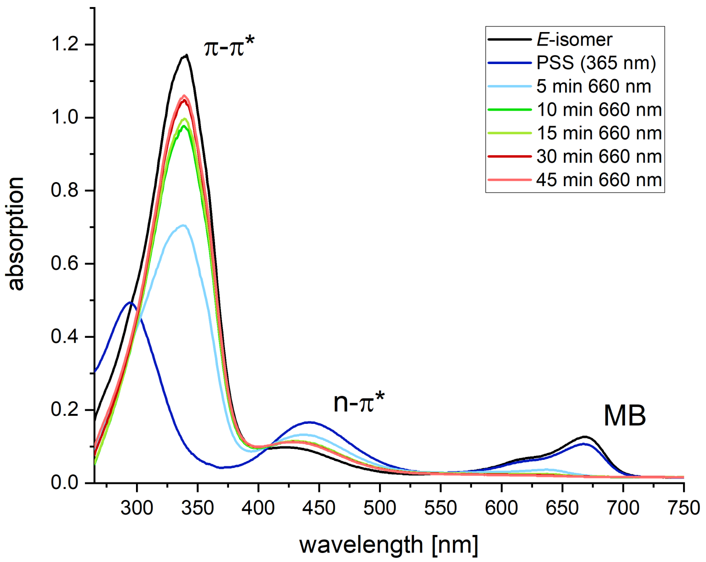

Figure S25: UV/Vis spectra of a 50 µM sample solution of PATP with 10mol% MB after several irradiation steps.

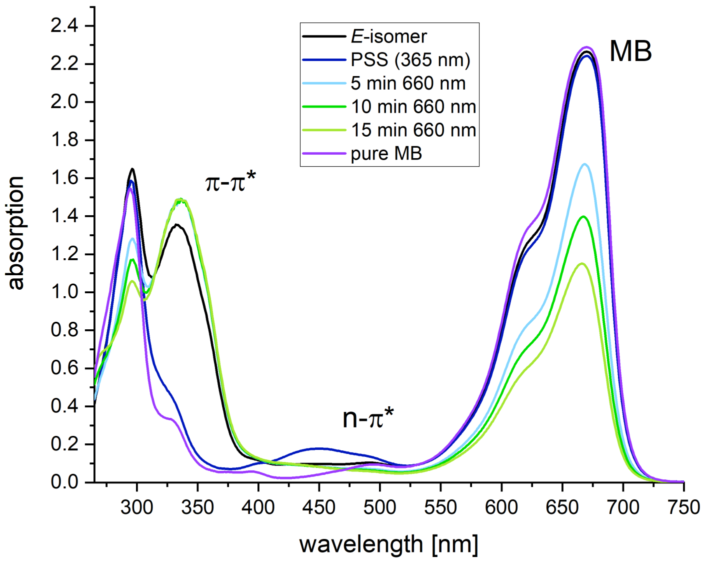

Figure S26: UV/Vis spectra of a 50 µM sample solution of PATP with 100mol% MB after several irradiation steps and a UV/Vis spectrum of a 50 µM (= 100mol%) solution of pure MB (purple curve) for comparison.

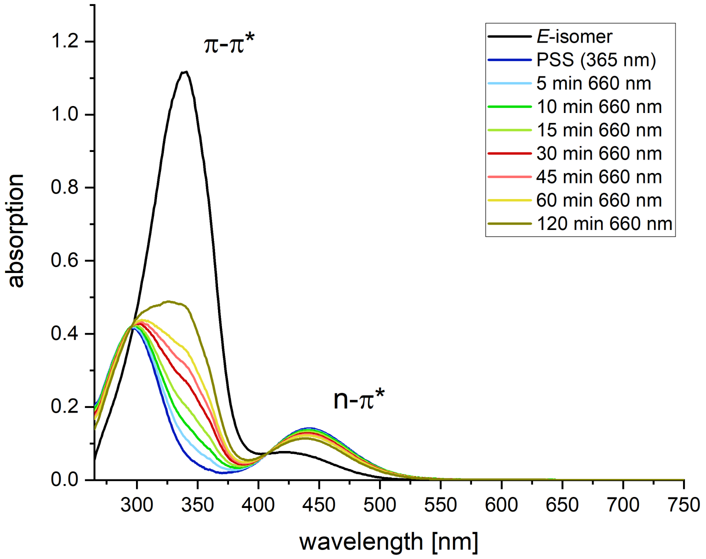

Figure S27: UV/Vis spectra of a 50 µM sample solution of PATP without any MB after several irradiation steps.

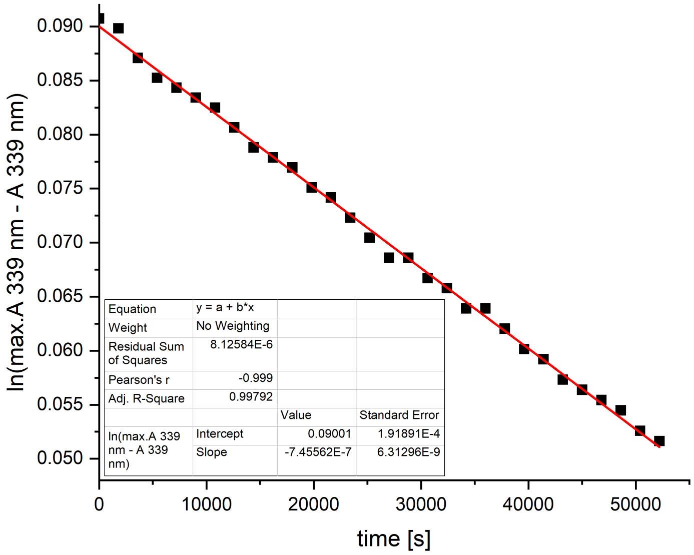

Figure S28: Experimental kinetic data for the thermal Z/E isomerization of PATP with 1mol% MB content of the sample solution.

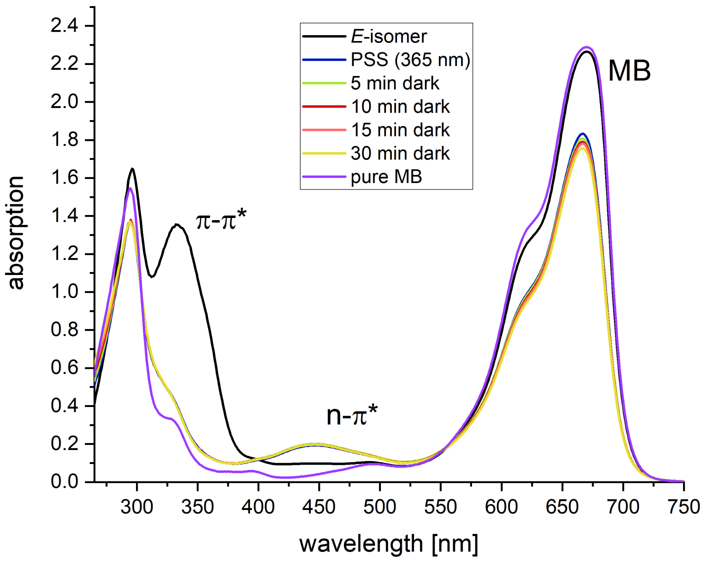

Figure S29: UV/Vis spectra of a 50 µM sample solution of PATP with 100mol% MB after initial irradiation with 365 nm towards the *Z*-isomer and short time intervals in the dark.

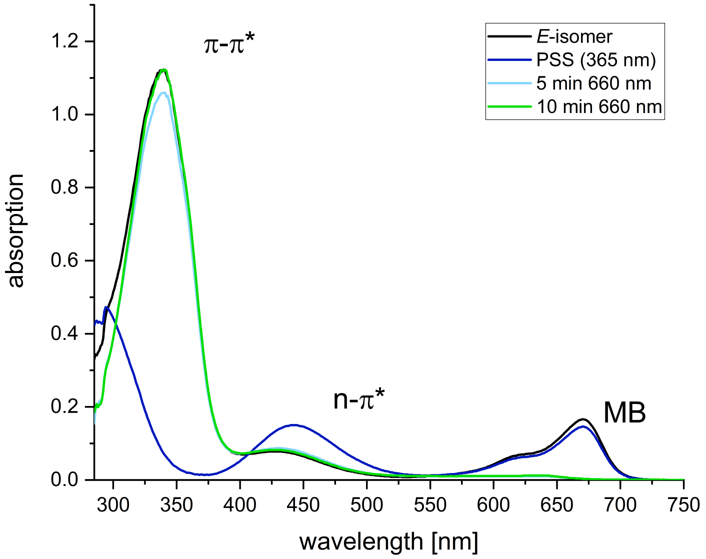

Figure S30: UV/Vis spectra of a 50 µM sample solution of PATP with 10mol% MB after several irradiation steps under oxygen-depleted conditions.

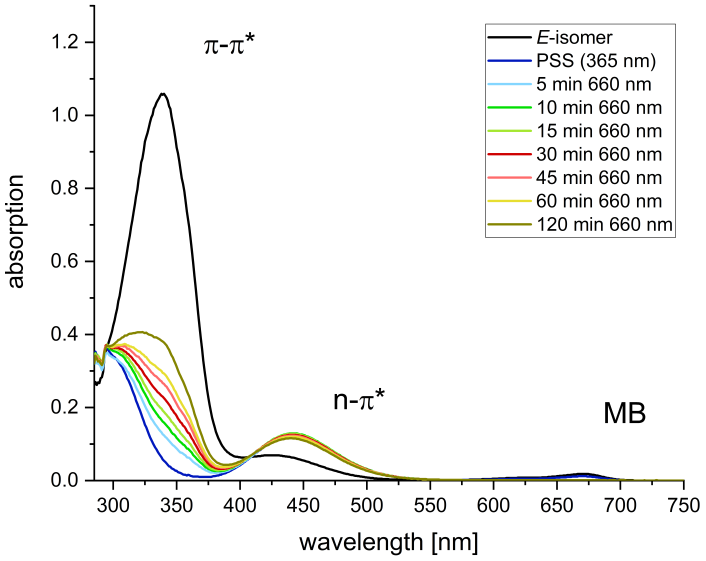

Figure S31: UV/Vis spectra of a 50 µM sample solution of PATP with 1mol% MB after several irradiation steps under oxygen-depleted conditions.

S5.4 ^{1}H-NMR and ^{13}C-NMR spectra

4-Hydroxy-3-(phenyldiazenyl)pent-3-en-2-one (1)

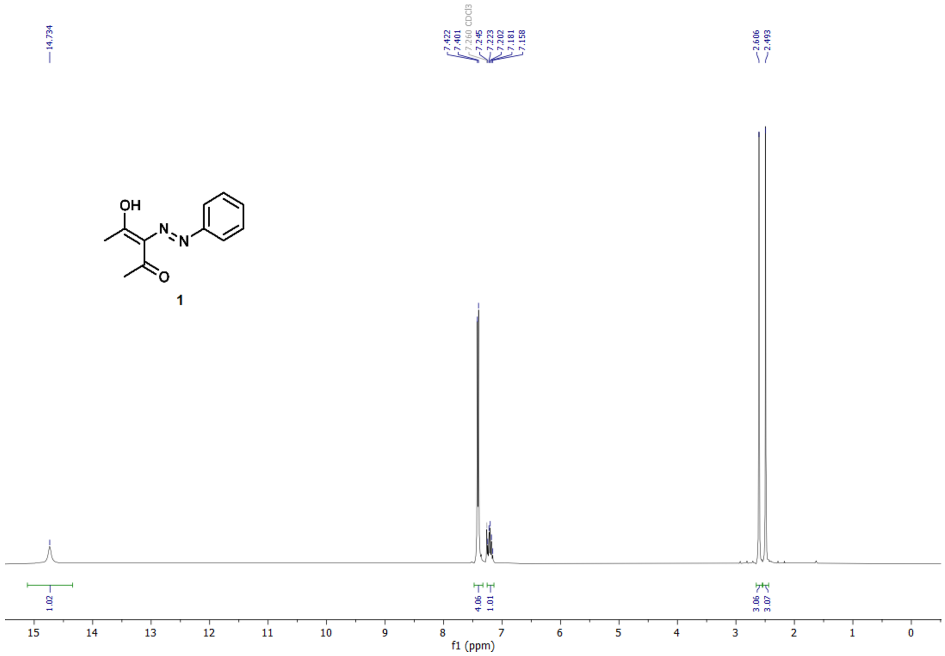

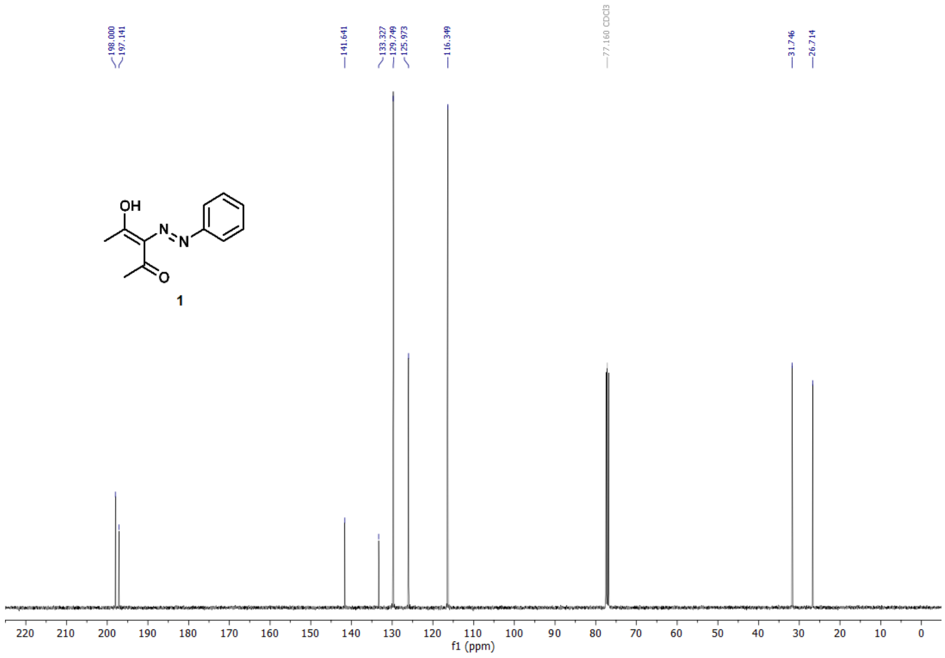

Phenylazo-1,3,5-trimethyl-pyrazole (2) - PATP

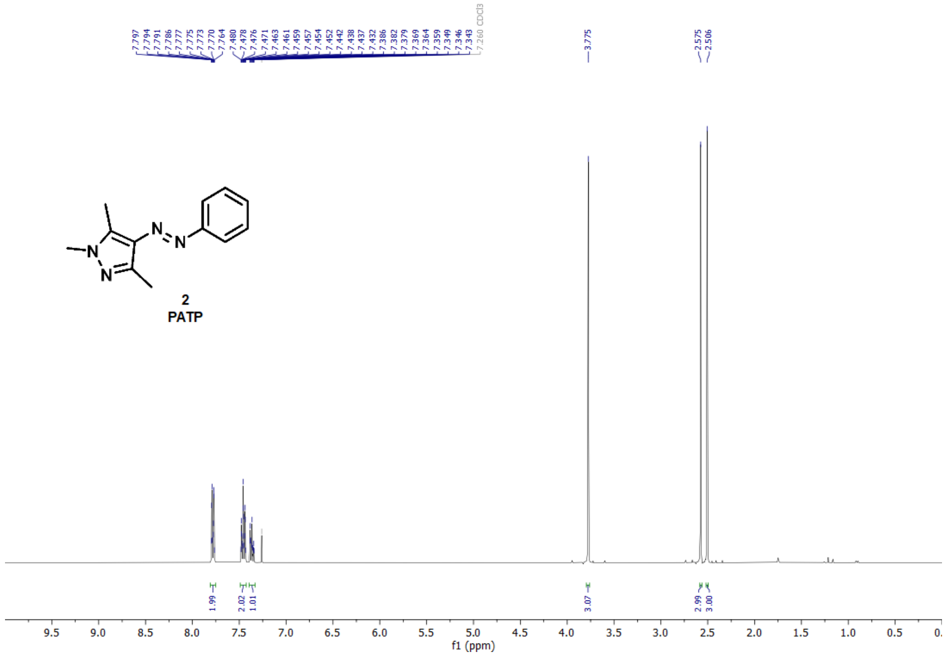

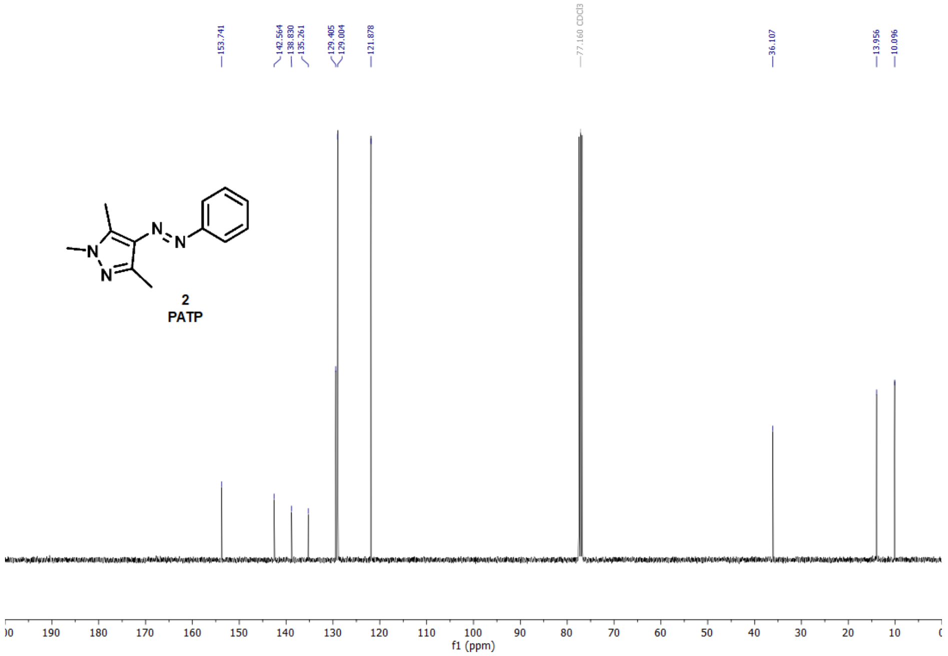

References

(1) Pracht, P.; Bohle, F.; Grimme, S. Automated Exploration of the Low-Energy Chemical Space with Fast Quantum Chemical Methods. *Phys. Chem. Chem. Phys.* **2020**, *22*, 7169–7192.

(2) Grimme, S. Exploration of Chemical Compound, Conformer, and Reaction Space with Meta-Dynamics Simulations Based on Tight-Binding Quantum Chemical Calculations. *J. Chem. Theor. Comput.* **2019**, *15*, 2847–2862.

(3) Chai, J.-D.; Head-Gordon, M. Long-Range Corrected Hybrid Density Functionals with Damped Atom–Atom Dispersion Corrections. *Phys. Chem. Chem. Phys.* **2008**, *10*, 6615.

(4) Grimme, S.; Antony, J.; Ehrlich, S.; Krieg, H. A Consistent and Accurate *Ab Initio* Parametrization of Density Functional Dispersion Correction (DFT-D) for the 94 Elements H-Pu. *J. Chem. Phys.* **2010**, *132*, 154104.

(5) Crespi, S.; Simeth, N. A.; Bellisario, A.; Fagnoni, M.; König, B. Unraveling the Thermal Isomerization Mechanisms of Heteroaryl Azoswitches: Phenylazoindoles as Case Study. *J. Phys. Chem.* **2019**, *123*, 1814–1823.

(6) Adrion, D. M.; Kaliakin, D. S.; Neal, P.; Lopez, S. A. Benchmarking of Density Functionals for Z-Azoarene Half-Lives via Automated Transition State Search. *J. Phys. Chem. A* **2021**, *125*, 6474–6485.

(7) Weigend, F.; Ahlrichs, R. Balanced Basis Sets of Split Valence, Triple Zeta Valence and Quadruple Zeta Valence Quality For H to Rn: Design and Assessment of Accuracy. *Phys. Chem. Chem. Phys.* **2005**, *7*, 3297.

(8) Weigend, F. Accurate Coulomb-Fitting Basis Sets For H to Rn. *Phys. Chem. Chem. Phys.* **2006**, *8*, 1057.

(9) Marenich, A. V.; Cramer, C. J.; Truhlar, D. G. Universal Solvation Model Based on Solute Electron Density and on a Continuum Model of the Solvent Defined by the Bulk Dielectric Constant and Atomic Surface Tensions. *J. Phys. Chem. B* **2009**, *113*, 6378–6396.

(10) Frisch, M. J.; Trucks, G. W.; Schlegel, H. B.; Scuseria, G. E.; Robb, M. A.; Cheeseman, J. R.; Scalmani, G.; Barone, V.; Petersson, G. A.; Nakatsuji, H. et al. Gaussian 16 Revision C.01. 2016; Gaussian Inc. Wallingford CT.

(11) Hirata, S.; Head-Gordon, M. Time-Dependent Density Functional Theory within the Tamm–Dancoff Approximation. *Chem. Phys. Lett.* **1999**, *314*, 291–299.

(12) Axelrod, S.; Shakhnovich, E.; Gómez-Bombarelli, R. Thermal Half-Lives of Azobenzene Derivatives: Virtual Screening Based on Intersystem Crossing Using a Machine Learning Potential. *ACS Cent. Sci.* **2023**, *9*, 166–176.

(13) Neese, F.; Wennmohs, F.; Becker, U.; Riplinger, C. The ORCA Quantum Chemistry Program Package. *J. Chem. Phys.* **2020**, *152*, 224108.

(14) Neese, F. Software Update: The ORCA Program System—Version 5.0. *WIREs Comput. Mol. Sci.* **2022**, *12*, e1606.

(15) Ekström, U.; Visscher, L.; Bast, R.; Thorvaldsen, A. J.; Ruud, K. Arbitrary-Order Density Functional Response Theory from Automatic Differentiation. *J. Chem. Theor. Comput.* **2010**, *6*, 1971–1980.

(16) Casanova, D.; Krylov, A. I. Spin-Flip Methods in Quantum Chemistry. *Phys. Chem. Chem. Phys.* **2020**, *22*, 4326–4342.

(17) Mai, S.; Richter, M.; Heindl, M.; Menger, M. F. S. J.; Atkins, A.; Ruckenbauer, M.; Plasser, F.; Ibele, L. M.; Kropf, S.; Oppel, M. et al. SHARC2.1: Surface Hopping

Including Arbitrary Couplings — Program Package for Non-Adiabatic Dynamics. sharc-md.org, 2019.

(18) Perdew, J. P.; Ernzerhof, M.; Burke, K. Rationale for Mixing Exact Exchange with Density Functional Approximations. *J. Chem. Phys.* **1996**, *105*, 9982–9985.

(19) Adamo, C.; Barone, V. Toward Reliable Density Functional Methods without Adjustable Parameters: The PBE0 Model. *J. Chem. Phys.* **1999**, *110*, 6158–6170.

(20) Grimme, S.; Ehrlich, S.; Goerigk, L. Effect of the Damping Function in Dispersion Corrected Density Functional Theory. *J. Comput. Chem.* **2011**, *32*, 1456–1465.

(21) Zhao, Y.; Truhlar, D. G. Density Functional for Spectroscopy: No Long-Range Self-Interaction Error, Good Performance for Rydberg and Charge-Transfer States, and Better Performance on Average than B3LYP for Ground States. *The Journal of Physical Chemistry A* **2006**, *110*, 13126–13130.

(22) Grimme, S. Semiempirical Hybrid Density Functional with Perturbative Second-Order Correlation. *J. Chem. Phys.* **2006**, *124*, 034108.

(23) Møller, C.; Plesset, M. S. Note on an Approximation Treatment for Many-Electron Systems. *Phys. Rev.* **1934**, *46*, 618–622.

(24) Goerigk, L.; Grimme, S. Efficient and Accurate Double-Hybrid-Meta-GGA Density Functionals—Evaluation with the Extended GMTKN30 Database for General Main Group Thermochemistry, Kinetics, and Noncovalent Interactions. *J. Chem. Theor. Comput.* **2011**, *7*, 291–309.

(25) Weigend, F.; Häser, M. RI-MP2: First Derivatives and Global Consistency. *Theor. Chem. Acta* **1997**, *97*, 331–340.

(26) Weigend, F.; Häser, M.; Patzelt, H.; Ahlrichs, R. RI-MP2: Optimized Auxiliary Basis Sets and Demonstration of Efficiency. *Chem. Phys. Lett.* **1998**, *294*, 143–152.

(27) Bernholdt, D. E.; Harrison, R. J. Large-Scale Correlated Electronic Structure Calculations: The RI-MP2 Method on Parallel Computers. *Chem. Phys. Lett.* **1996**, *250*, 477–484.

(28) Feyereisen, M.; Fitzgerald, G.; Komornicki, A. Use of Approximate Integrals In Ab Initio Theory. An Application in MP2 Energy Calculations. *Chem. Phys. Lett.* **1993**, *208*, 359–363.

(29) Cammi, R.; Mennucci, B.; Tomasi, J. Fast Evaluation of Geometries and Properties of Excited Molecules in Solution: A Tamm-Dancoff Model with Application to 4-Dimethylaminobenzonitrile. *J. Phys. Chem.* **2000**, *104*, 5631–5637.

(30) Hellweg, A.; Hättig, C.; Höfener, S.; Klopper, W. Optimized Accurate Auxiliary Basis Sets for RI-MP2 and RI-CC2 Calculations For the Atoms Rb to Rn. *Theor. Chem. Acc.* **2007**, *117*, 587–597.

(31) Barone, V.; Cossi, M. Quantum Calculation of Molecular Energies and Energy Gradients in Solution by a Conductor Solvent Model. *J. Phys. Chem.* **1998**, *102*, 1995–2001.

(32) Cossi, M.; Rega, N.; Scalmani, G.; Barone, V. Polarizable Dielectric Model of Solvation with Inclusion of Charge Penetration Effects. *J. Chem. Phys.* **2001**, *114*, 5691–5701.

(33) Roos, B. O.; Lindh, R.; Malmqvist, P.-Å.; Veryazov, V.; Widmark, P.-O. Main Group Atoms and Dimers Studied with a New Relativistic ANO Basis Set. *J. Phys. Chem.* **2003**, *108*, 2851–2858.

(34) Aquilante, F.; Lindh, R.; Bondo Pedersen, T. Unbiased Auxiliary Basis Sets For Accurate Two-Electron Integral Approximations. *J. Chem. Phys.* **2007**, *127*, 114107.

(35) Aquilante, F.; Autschbach, J.; Baiardi, A.; Battaglia, S.; Borin, V. A.; Chibotaru, L. F.; Conti, I.; De Vico, L.; Delcey, M.; Fdez. Galván, I. et al. Modern Quantum Chemistry with [Open]Molcas. *J. Chem. Phys.* **2020**, *152*, 214117.

(36) Forsberg, N.; Malmqvist, P.-Å. Multiconfiguration Perturbation Theory with Imaginary Level Shift. *Chem. Phys. Lett.* **1997**, *274*, 196–204.

(37) Ghigo, G.; Roos, B. O.; Malmqvist, P.-Å. A Modified Definition of the Zeroth-Order Hamiltonian in Multiconfigurational Perturbation Theory CASPT2. *Chem. Phys. Lett.* **2004**, *396*, 142–149.

(38) Andersson, K.; Malmqvist, P.-Å.; Roos, B. O. Second-Order Perturbation Theory with a Complete Actice Space Self-Consistent Field Reference Function. *J. Chem. Phys.* **1992**, *96*, 1218–1226.

(39) Andersson, K.; Roos, B. O. Multiconfigurational Second-Order Perturbation Theory: A Test of Geometries and Binding Energies. *Int. J. Quant. Chem.* **1993**, *45*, 591–607.

(40) Andersson, K. Different Forms of the Zeroth-Order Hamiltonian in Second-Order Perturbation Theory with a Complete Actice Space Self-Consistent Field Reference Function. *Theor. Chim. Acta* **1995**, *91*, 31–46.

(41) Zobel, J. P.; Nogueira, J. J.; González, L. The IPEA Dilemma in CASPT2. *Chem. Sci.* **2017**, *8*, 1482–1499.

(42) Li Manni, G.; Carlson, R. K.; Luo, S.; Ma, D.; Olsen, J.; Truhlar, D. G.; Gagliardi, L. Multiconfigurational Pair-Density Functional Theory. *J. Chem. Theory Comput.* **2014**, *10*, 3669–3680.

(43) Pandharkar, R.; Hermes, M. R.; Truhlar, D. G.; Gagliardi, L. A New Mixing of Nonlocal Exchange and Nonlocal Correlation with Multiconfigurational Pair-Density Functional Theory. *J. Phys. Chem. Lett.* **2020**, *11*, 10158–10163.

(44) Eyring, H. The Activated Complex in Chemical Reactions. *J. Chem. Phys.* **1935**, *3*, 107–115.

(45) Truhlar, D. G.; Garrétt, B. C.; Klippenstein, S. J. Current Status of Transition-State Theory. *J. Phys. Chem.* **1996**, *100*, 12771–12800.

(46) Lykhin, A. O.; Kaliakin, D. S.; dePolo, G. E.; Kuzubov, A. A.; Varganov, S. A. Nonadiabatic Transition State Theory: Application to Intersystem Crossings in the Active Sites of Metal-Sulfur Proteins. *Int. J. Quant. Chem.* **2016**, *116*, 750–761.

(47) Reimann, M.; Teichmann, E.; Hecht, S.; Kaupp, M. Solving the Azobenzene Entropy Puzzle: Direct Evidence for Multi-State Reactivity. *J. Phys. Chem. Lett.* **2022**, *13*, 10882–10888.

(48) Liu, S.; Srinivasan, S.; Tao, J.; Grady, M. C.; Soroush, M.; Rappe, A. M. Modeling Spin-Forbidden Monomer Self-Initiation Reactions in Spontaneous Free-Radical Polymerization of Acrylates and Methacrylates. *J. Phys. Chem.* **2014**, *118*, 9310–9318.

(49) Wigner, E. Über das Überschreiten von Potentialschwellen bei Chemischen Reaktionen. *Zeitschrift für Physikalische Chemie* **1932**, *19B*, 203–216.

(50) Canneaux, S.; Bohr, F.; Henon, E. KiSThelP: A Program to Predict Thermodynamic Properties and Rate Constants From Quantum Chemistry Results. *J. Comput. Chem.* **2014**, *35*, 82–93.

(51) Wang, Y. P.; Zhang, Z. X.; Xie, M.; Bai, F. Q.; Wang, P. X.; Zhang, H. X. Theoretical Study on Thermal *cis*-to-*trans* Isomerization of BF_2-Coordinated Azo Compounds of the para-Substitution with Electron Donating Groups. *Dyes Pigm.* **2016**, *129*, 100–108.

(52) Hüll, K.; Morstein, J.; Trauner, D. *In Vivo* Photopharmacology. *Chem. Rev.* **2018**, *118*, 10710–10747.

(53) Fischer, E. Calculation of Photostationary States in Systems A $\rightleftarrows$ B When Only A Is Known. *J. Phys. Chem.* **1967**, *71*, 3704–3706.

Appendix

A1 XYZ Coordinates - DFT

```
Z
C +2.283690 +0.565150 +0.121710
C +1.007440 +0.530000 -0.428270
C +0.788910 -0.826440 -0.791280
N +2.720220 -0.698800 +0.120820
N +1.845320 -1.546000 -0.456850
C +4.000500 -1.183790 +0.582300
H +3.909640 -2.249370 +0.777360
H +4.771650 -1.016100 -0.171330
H +4.277460 -0.672020 +1.502500
C -0.346020 -1.461990 -1.515110
H -0.729390 -0.803670 -2.295350
H -0.011470 -2.391910 -1.975050
H -1.173730 -1.689090 -0.839810
C +3.063800 +1.704500 +0.662150
H +3.100050 +1.673340 +1.753760
H +4.089710 +1.687090 +0.290940
H +2.597330 +2.642840 +0.365140
N +0.308110 +1.723350 -0.668090
N -0.922580 +1.855870 -0.591890
C -1.788820 +0.852530 -0.064300
C -1.574620 +0.288160 +1.189450
C -2.949180 +0.567420 -0.772830
C -2.507950 -0.595120 +1.707070
H -0.689510 +0.543870 +1.757380
C -3.866170 -0.334500 -0.257830
H -3.116300 +1.043400 -1.731510
C -3.646870 -0.920250 +0.981530
H -2.343620 -1.032660 +2.684070
H -4.759250 -0.572960 -0.822030
H -4.368380 -1.617660 +1.388440
```

```
M_1
C +2.703365 +0.425228 -0.473910
C +1.511780 +0.565028 +0.243289
C +1.432390 -0.593512 +1.072047
N +3.228222 -0.740261 -0.082364
N +2.481427 -1.363064 +0.856339
C +4.486384 -1.313693 -0.498671
H +4.625924 -1.160753 -1.567586
H +5.316421 -0.855770 +0.042092
H +4.459167 -2.379892 -0.288515
C +0.383009 -0.968704 +2.057335
H -0.512339 -1.347583 +1.558736
H +0.758624 -1.744425 +2.724658
H +0.083395 -0.105658 +2.654350
C +3.326148 +1.319860 -1.477223
H +2.747755 +2.238587 -1.561601
H +4.348985 +1.576418 -1.193420
H +3.362630 +0.842668 -2.459376
N +0.690691 +1.645366 +0.138459
N -0.470278 +1.731789 +0.679739
C -1.531647 +0.978664 +0.207630
C -1.466432 +0.173766 -0.939004
C -2.723196 +1.038395 +0.942657
C -2.570742 -0.567780 -1.318698
H -0.558545 +0.141574 -1.526918
C -3.815277 +0.287930 +0.554748
H -2.762421 +1.669376 +1.822210
C -3.744187 -0.520529 -0.575576
H -2.518414 -1.186031 -2.206624
H -4.730014 +0.331078 +1.132946
H -4.603124 -1.105471 -0.879748
```

TS_r

C +2.754136 -0.693377 -0.161483

C +1.538855 -0.100878 -0.598667

C +1.592143 +1.245040 -0.066073

N +3.385742 +0.238276 +0.541572

N +2.704704 +1.415869 +0.606658

C +4.645104 +0.102335 +1.240105

H +4.548211 -0.609971 +2.060364

H +5.420322 -0.239841 +0.555040

H +4.910076 +1.079288 +1.633797

C +0.601775 +2.344987 -0.217733

H -0.325110 +2.116125 +0.313225

H +1.014445 +3.270104 +0.182661

H +0.343993 +2.495230 -1.267249

C +3.253116 -2.071561 -0.364820

H +2.671179 -2.558499 -1.145380

H +4.305745 -2.074922 -0.653104

H +3.155623 -2.659103 +0.552288

N +0.633947 -0.726087 -1.321931

N -0.469824 -0.110413 -1.700785

C -1.569951 -0.200987 -0.886447

C -1.594176 -0.937289 +0.314671

C -2.720415 +0.508796 -1.280297

C -2.734635 -0.943513 +1.093881

H -0.717530 -1.499353 +0.609677

C -3.847080 +0.502092 -0.483899

H -2.690354 +1.070031 -2.206708

C -3.861457 -0.222562 +0.706893

H -2.750532 -1.515832 +2.014019

H -4.723420 +1.063160 -0.786146

H -4.748533 -0.228444 +1.328472

T_min

C +2.444400 -0.742620 -0.193690

C +1.219800 -0.100420 -0.376780

C +1.419280 +1.232250 +0.081840

N +3.263040 +0.179260 +0.331480

N +2.664570 +1.377970 +0.495360

C +4.663230 +0.019300 +0.644820

H +4.824250 -0.934350 +1.145820

H +5.269250 +0.056760 -0.262100

H +4.955580 +0.828570 +1.309130

C +0.439410 +2.350460 +0.134320

H -0.406580 +2.101940 +0.779060

H +0.919920 +3.247810 +0.524400

H +0.039610 +2.572620 -0.857080

C +2.849250 -2.138200 -0.485240

H +2.026730 -2.666110 -0.965980

H +3.715820 -2.166950 -1.149190

H +3.112350 -2.672280 +0.430910

N +0.100400 -0.701410 -0.860840

N -0.947000 -0.052180 -1.235860

C -2.113220 -0.176030 -0.502310

C -2.209810 -0.928830 +0.676290

C -3.235760 +0.511820 -0.980370

C -3.410460 -0.979490 +1.359200

H -1.344160 -1.469160 +1.037620

C -4.428210 +0.456270 -0.285080

H -3.147250 +1.086880 -1.893990

C -4.520910 -0.289120 +0.885460

H -3.484380 -1.562340 +2.269070

H -5.292240 +0.993780 -0.655450

H -5.457910 -0.335020 +1.426100

M_2

C -2.812327 -0.745318 +0.172586

C -1.634730 -0.034058 +0.407260

C -1.863551 +1.264053 -0.132442

N -3.632398 +0.106950 -0.456190

N -3.081157 +1.326060 -0.638347

C -4.995628 -0.137756 -0.862631

H -5.082208 -1.138442 -1.283324

H -5.676202 -0.043545 -0.014548

H -5.260210 +0.594236 -1.621562

C -0.943476 +2.432571 -0.163189

H -0.034883 +2.205871 -0.725157

H -1.438322 +3.282600 -0.633270

H -0.639583 +2.720178 +0.845362

C -3.168442 -2.146273 +0.500028

H -2.361649 -2.608344 +1.067330

H -4.083400 -2.192529 +1.094265

H -3.329665 -2.732223 -0.408020

N -0.530027 -0.555325 +1.002129

N +0.482861 +0.163019 +1.348050

C +1.690885 -0.036431 +0.706876

C +1.872190 -0.975490 -0.318611

C +2.769410 +0.757517 +1.118454

C +3.111670 -1.106655 -0.915817

H +1.040484 -1.596946 -0.625176

C +4.000323 +0.621665 +0.506526

H +2.616339 +1.478263 +1.912483

C +4.177770 -0.311446 -0.509982

H +3.250964 -1.836699 -1.703758

H +4.828872 +1.242928 +0.823270

H +5.145392 -0.421431 -0.982297

E

C +2.315960 -0.882620 +0.027320

C +1.284290 +0.053870 -0.005660

C +1.923660 +1.325070 -0.054030

N +3.448330 -0.178080 -0.006600

N +3.230270 +1.158790 -0.049860

C +4.800320 -0.679670 +0.071250

H +5.460100 +0.040200 -0.407150

H +5.100990 -0.813980 +1.111650

H +4.869120 -1.632420 -0.450080

C +1.331460 +2.687310 -0.111260

H +0.703510 +2.877010 +0.760960

H +2.125160 +3.434070 -0.147320

H +0.696520 +2.799880 -0.991800

C +2.271150 -2.362930 +0.092460

H +2.751710 -2.805680 -0.782650

H +2.790810 -2.728860 +0.980370

H +1.236890 -2.699370 +0.129040

N -0.039880 -0.329320 +0.008730

N -0.887350 +0.583810 -0.013450

C -2.238090 +0.143830 -0.007800

C -2.637140 -1.183570 -0.162610

C -3.194420 +1.141110 +0.148990

C -3.984390 -1.498530 -0.148580

H -1.892840 -1.956780 -0.297780

C -4.542860 +0.818200 +0.166910

H -2.867060 +2.168000 +0.259770

C -4.940710 -0.502230 +0.018550

H -4.294470 -2.529080 -0.271970

H -5.282350 +1.599040 +0.294170

H -5.993150 -0.757380 +0.027850

TS_iAr

C +2.671800 -0.696850 +0.011460

C +1.324600 -0.346340 +0.008270

C +1.288420 +1.073150 +0.006760

N +3.339480 +0.457250 +0.017950

N +2.519260 +1.538410 +0.009220

C +4.769880 +0.651530 -0.037450

H +5.103020 +0.742850 -1.072300

H +5.015650 +1.561230 +0.505620

H +5.270470 -0.193090 +0.430720

C +0.114320 +1.984380 +0.008640

H -0.510200 +1.815680 +0.888670

H +0.449390 +3.021430 +0.008760

H -0.512210 +1.816550 -0.870080

C +3.321160 -2.029150 +0.003080

H +3.968540 -2.139150 -0.868970

H +3.933860 -2.167240 +0.896300

H +2.563120 -2.809450 -0.023110

N +0.315380 -1.306520 +0.010660

N -0.842950 -0.931370 +0.008790

C -2.097840 -0.535580 +0.002270

C -2.790820 -0.309570 +1.216560

C -2.780300 -0.318850 -1.219740

C -4.109340 +0.101010 +1.185790

H -2.274580 -0.462450 +2.155150

C -4.099010 +0.091870 -1.203500

H -2.255960 -0.478920 -2.152620

C -4.784880 +0.307800 -0.012610

H -4.619200 +0.266530 +2.128310

H -4.600710 +0.250110 -2.151640

H -5.817300 +0.631110 -0.018310

TS_iPy

C -2.513760 -0.746120 +0.086510

C -1.217130 -0.178540 -0.016240

C -1.462940 +1.227590 -0.215700

N -3.366070 +0.256110 -0.025330

N -2.749830 +1.462200 -0.230150

C -4.809440 +0.216470 -0.008060

H -5.177790 +1.022540 +0.624140

H -5.198280 +0.339910 -1.019380

H -5.139940 -0.737830 +0.393900

C -0.475360 +2.318740 -0.412840

H +0.312060 +2.009380 -1.100450

H -0.967700 +3.205850 -0.810460

H +0.000390 +2.585360 +0.533560

C -2.887330 -2.161510 +0.288160

H -1.982680 -2.768220 +0.306890

H -3.420240 -2.296960 +1.231570

H -3.533510 -2.514150 -0.517710

N -0.086210 -0.848270 +0.042380

N +0.975990 -1.484390 +0.044320

C +2.191270 -0.708320 +0.054820

C +3.310120 -1.287940 -0.530280

C +2.294430 +0.526700 +0.682600

C +4.520180 -0.611000 -0.530310

H +3.217810 -2.264930 -0.991300

C +3.509990 +1.192590 +0.697420

H +1.426390 +0.949040 +1.171920

C +4.622790 +0.631240 +0.083040

H +5.386580 -1.055420 -1.004910

H +3.592380 +2.151080 +1.195790

H +5.570400 +1.155540 +0.093160

A2 XYZ Coordinates - CASSCF

Z

```
C +2.194368 +0.535051 +0.137456
C +1.004404 +0.494385 -0.539370
C +0.855899 -0.862669 -0.964703
N +2.654053 -0.740685 +0.168859
N +1.868032 -1.569742 -0.528689
C +3.922527 -1.214958 +0.658155
H +3.843563 -2.281402 +0.810427
H +4.726555 -1.018640 -0.045573
H +4.159721 -0.742921 +1.603161
C -0.200163 -1.493855 -1.815826
H -0.502572 -0.829673 -2.618799
H +0.191243 -2.404744 -2.251818
H -1.083192 -1.740997 -1.238038
C +2.907381 +1.686834 +0.763153
H +2.898813 +1.623809 +1.847999
H +3.942707 +1.730576 +0.439438
H +2.424064 +2.611225 +0.474273
N +0.291425 +1.672977 -0.888365
N -0.932653 +1.808168 -0.730249
C -1.760127 +0.841785 -0.079818
C -1.497062 +0.353620 +1.198169
C -2.962696 +0.533368 -0.704820
C -2.421716 -0.476916 +1.817185
H -0.591653 +0.623093 +1.705999
C -3.868029 -0.317067 -0.086000
H -3.177502 +0.958835 -1.667667
C -3.601371 -0.822212 +1.168413
H -2.219897 -0.852821 +2.803660
H -4.786634 -0.568296 -0.584772
H -4.309518 -1.470938 +1.651250
```

M_1

```
C +2.682959 +0.396952 -0.507433
C +1.505662 +0.523527 +0.207670
C +1.473514 -0.630889 +1.075041
N +3.245996 -0.770018 -0.107282
N +2.531568 -1.363739 +0.863586
C +4.561397 -1.268294 -0.411788
H +4.783233 -1.119220 -1.460675
H +5.327912 -0.779426 +0.183395
H +4.575878 -2.327286 -0.198985
C +0.472143 -1.020573 +2.115223
H -0.463728 -1.339454 +1.670309
H +0.872032 -1.838857 +2.700892
H +0.255971 -0.189320 +2.777572
C +3.280153 +1.299907 -1.531576
H +2.663879 +2.183160 -1.633512
H +4.280540 +1.614027 -1.248123
H +3.344524 +0.815173 -2.501855
N +0.680958 +1.602917 +0.091666
N -0.479013 +1.660859 +0.670872
C -1.542704 +0.933647 +0.196820
C -1.522331 +0.161190 -0.975659
C -2.739621 +1.020450 +0.938334
C -2.667580 -0.519278 -1.372956
H -0.630329 +0.105313 -1.567861
C -3.853665 +0.340123 +0.541101
H -2.746834 +1.630242 +1.822470
C -3.833252 -0.440468 -0.619105
H -2.648311 -1.107230 -2.272638
H -4.755818 +0.408412 +1.121909
H -4.715432 -0.969249 -0.929410
```

TS_r

C +2.718895 -0.706237 -0.139466

C +1.536152 -0.107221 -0.561524

C +1.608773 +1.244223 -0.040572

N +3.389775 +0.234421 +0.569873

N +2.721465 +1.398795 +0.620769

C +4.661436 +0.108830 +1.231557

H +4.624928 -0.630909 +2.025118

H +5.443579 -0.166663 +0.532012

H +4.899749 +1.070321 +1.660824

C +0.653688 +2.385734 -0.193438

H -0.280029 +2.197979 +0.325168

H +1.104286 +3.279167 +0.220762

H +0.420589 +2.562926 -1.237616

C +3.232249 -2.083766 -0.380219

H +2.530681 -2.624315 -1.001539

H +4.192446 -2.065573 -0.888494

H +3.358627 -2.631983 +0.549737

N +0.628323 -0.743988 -1.328642

N -0.489464 -0.107475 -1.679750

C -1.564984 -0.194629 -0.861345

C -1.604419 -0.917426 +0.350560

C -2.738198 +0.486557 -1.288823

C -2.771640 -0.936358 +1.105640

H -0.740074 -1.459904 +0.678536

C -3.866291 +0.463820 -0.536047

H -2.699275 +1.019665 -2.220363

C -3.902321 -0.248809 +0.677651

H -2.797818 -1.491410 +2.025843

H -4.743420 +0.989209 -0.868591

H -4.801604 -0.266281 +1.264978

T_min

C +2.375876 -0.763625 -0.183739

C +1.179230 -0.117512 -0.446242

C +1.373814 +1.230865 +0.036575

N +3.169294 +0.145718 +0.435922

N +2.571605 +1.346340 +0.537765

C +4.571609 +0.036729 +0.740026

H +4.794813 -0.954125 +1.113856

H +5.191969 +0.232851 -0.130495

H +4.806791 +0.760334 +1.507186

C +0.436662 +2.396188 +0.015283

H -0.441854 +2.210313 +0.623949

H +0.947874 +3.268199 +0.403933

H +0.098941 +2.606884 -0.993343

C +2.785289 -2.167802 -0.469274

H +1.992092 -2.669880 -1.007211

H +3.684382 -2.203543 -1.077836

H +2.978938 -2.720348 +0.446150

N +0.120614 -0.724852 -1.039049

N -0.953947 -0.033337 -1.326068

C -2.060312 -0.169765 -0.537275

C -2.117429 -0.964430 +0.621146

C -3.212659 +0.547632 -0.933207

C -3.294476 -1.025539 +1.359014

H -1.256712 -1.527634 +0.925088

C -4.357141 +0.481503 -0.197546

H -3.158445 +1.143755 -1.825184

C -4.415376 -0.306104 +0.960324

H -3.334638 -1.636582 +2.242589

H -5.224813 +1.035141 -0.508486

H -5.322992 -0.356194 +1.533069

M_2

C -2.775042 -0.773610 +0.169991

C -1.608843 -0.073459 +0.429120

C -1.824453 +1.231744 -0.152587

N -3.571723 +0.066214 -0.537305

N -3.005866 +1.275410 -0.701331

C -4.958281 -0.108563 -0.879725

H -5.132974 -1.119779 -1.224122

H -5.613406 +0.094397 -0.036728

H -5.194601 +0.578229 -1.679378

C -0.919322 +2.421655 -0.183875

H -0.010196 +2.213002 -0.737895

H -1.433989 +3.245565 -0.662371

H -0.632107 +2.720193 +0.818030

C -3.152734 -2.168451 +0.533428

H -2.365662 -2.608734 +1.130997

H -4.072480 -2.193460 +1.110746

H -3.295553 -2.785243 -0.349641

N -0.550912 -0.606162 +1.090382

N +0.473424 +0.149792 +1.394953

C +1.654207 -0.036558 +0.734030

C +1.829808 -0.942039 -0.336219

C +2.748913 +0.737788 +1.156963

C +3.047124 -1.050961 -0.948407

H +1.004019 -1.546455 -0.657746

C +3.975911 +0.618035 +0.522453

H +2.611302 +1.418649 +1.975986

C +4.132191 -0.276328 -0.533723

H +3.170849 -1.745202 -1.760233

H +4.807275 +1.215340 +0.849882

H +5.082424 -0.374012 -1.025373

E

C +2.318282 -0.893978 -0.024232

C +1.287793 +0.011895 -0.012544

C +1.915966 +1.303892 -0.018108

N +3.459504 -0.168704 -0.054997

N +3.213636 +1.152662 -0.031891

C +4.815983 -0.631382 +0.078790

H +5.469336 +0.119407 -0.341508

H +5.087719 -0.791684 +1.118480

H +4.950558 -1.556722 -0.466067

C +1.314869 +2.673001 -0.016260

H +0.689721 +2.824084 +0.856157

H +2.110768 +3.407893 -0.014297

H +0.691616 +2.827721 -0.889585

C +2.293955 -2.385627 -0.019703

H +2.755294 -2.788966 -0.916377

H +2.824149 -2.786364 +0.839274

H +1.270234 -2.730632 +0.020285

N -0.049580 -0.386557 -0.007642

N -0.880509 +0.537485 -0.004816

C -2.240099 +0.121092 +0.001370

C -2.664547 -1.210238 -0.003993

C -3.178717 +1.144597 +0.012700

C -4.021030 -1.492945 +0.002466

H -1.941793 -2.001064 -0.013282

C -4.537127 +0.848431 +0.019379

H -2.836561 +2.162764 +0.016118

C -4.957996 -0.460913 +0.014178

H -4.351405 -2.515961 -0.001905

H -5.256260 +1.647487 +0.028215

H -6.008221 -0.690985 +0.019215

A3 XYZ Coordinates - CASPT2

Z

C +2.276343 +0.575838 +0.144493
C +0.986570 +0.550642 -0.408324
C +0.753692 -0.808551 -0.763186
N +2.692817 -0.709241 +0.158294
N +1.810485 -1.565037 -0.414813
C +3.982562 -1.210200 +0.593517
H +3.872842 -2.278557 +0.779605
H +4.744722 -1.051234 -0.176453
H +4.284921 -0.704728 +1.513489
C -0.386594 -1.444584 -1.489349
H -0.745209 -0.801267 -2.297944
H -0.049677 -2.392228 -1.917062
H -1.231702 -1.640271 -0.821658
C +3.076382 +1.723329 +0.655571
H +3.117573 +1.740472 +1.751146
H +4.104085 +1.693039 +0.278436
H +2.607740 +2.650879 +0.317817
N +0.323540 +1.772183 -0.671708
N -0.935405 +1.902559 -0.614549
C -1.771711 +0.867712 -0.083247
C -1.528812 +0.273367 +1.164503
C -2.960499 +0.592122 -0.774521
C -2.451768 -0.641156 +1.683583
H -0.631182 +0.524073 +1.717678
C -3.861995 -0.337664 -0.268985
H -3.149168 +1.105591 -1.712330
C -3.610393 -0.966730 +0.965530
H -2.263053 -1.102250 +2.647881
H -4.767883 -0.569197 -0.820808
H -4.319254 -1.683722 +1.366481

M_1

C +2.719099 +0.438785 -0.496309
C +1.494800 +0.582723 +0.199559
C +1.394683 -0.585325 +1.025455
N +3.227367 -0.740595 -0.089864
N +2.460222 -1.376938 +0.838377
C +4.503355 -1.319625 -0.463197
H +4.676945 -1.174179 -1.531859
H +5.320439 -0.862027 +0.104119
H +4.456728 -2.385108 -0.238783
C +0.329665 -0.970549 +1.997741
H -0.596610 -1.250946 +1.484757
H +0.676131 -1.820204 +2.591398
H +0.092640 -0.136271 +2.665135
C +3.363920 +1.343817 -1.485118
H +2.779345 +2.264807 -1.544526
H +4.387936 +1.600271 -1.191894
H +3.400282 +0.894351 -2.484642
N +0.698253 +1.678928 +0.049209
N -0.456601 +1.743109 +0.667130
C -1.510076 +0.982853 +0.222094
C -1.458736 +0.149628 -0.932189
C -2.710568 +1.048087 +0.970539
C -2.573081 -0.600751 -1.293264
H -0.549276 +0.114311 -1.520983
C -3.816621 +0.282453 +0.595747
H -2.739292 +1.695464 +1.841220
C -3.756288 -0.546266 -0.530908
H -2.529593 -1.233511 -2.174285
H -4.727516 +0.334087 +1.184594
H -4.617854 -1.138779 -0.821253

TS_r

C +2.749987 -0.727245 -0.151742

C +1.527223 -0.142759 -0.581105

C +1.541078 +1.184274 -0.024350

N +3.365315 +0.218623 +0.583859

N +2.662653 +1.384015 +0.678999

C +4.643168 +0.104307 +1.258413

H +4.595914 -0.647067 +2.052979

H +5.427354 -0.170842 +0.547085

H +4.864582 +1.080193 +1.689976

C +0.525438 +2.271774 -0.148524

H -0.408784 +2.003046 +0.356259

H +0.920369 +3.185182 +0.303182

H +0.283710 +2.462264 -1.198438

C +3.303366 -2.080430 -0.421164

H +2.585701 -2.631432 -1.033727

H +4.253296 -2.029039 -0.966592

H +3.476066 -2.643049 +0.504064

N +0.634354 -0.808964 -1.345346

N -0.461172 -0.126469 -1.740501

C -1.538013 -0.191361 -0.918508

C -1.557765 -0.861687 +0.348176

C -2.712491 +0.489105 -1.350438

C -2.701065 -0.825743 +1.132642

H -0.671749 -1.393694 +0.673856

C -3.850366 +0.514714 -0.545020

H -2.687803 +0.986381 -2.314628

C -3.854069 -0.136005 +0.696267

H -2.711946 -1.335774 +2.090718

H -4.736666 +1.042072 -0.884076

H -4.741584 -0.115688 +1.320284

T_min

C +2.410136 -0.780465 -0.255175

C +1.169707 -0.157970 -0.506829

C +1.279494 +1.148116 +0.061921

N +3.137597 +0.132896 +0.423387

N +2.488116 +1.310905 +0.621495

C +4.517034 +0.007292 +0.850818

H +4.684955 -0.984140 +1.278641

H +5.202957 +0.161068 +0.010986

H +4.694041 +0.770097 +1.609075

C +0.262766 +2.240671 +0.102315

H -0.613610 +1.945908 +0.689505

H +0.707417 +3.130430 +0.554780

H -0.088318 +2.487053 -0.904536

C +2.886828 -2.141648 -0.621152

H +2.117701 -2.628403 -1.225800

H +3.813014 -2.104371 -1.205660

H +3.069849 -2.761722 +0.264488

N +0.135110 -0.783446 -1.147118

N -0.945614 -0.123693 -1.457492

C -2.038820 -0.181885 -0.621094

C -2.016778 -0.819990 +0.648427

C -3.217598 +0.467127 -1.052454

C -3.149913 -0.790338 +1.455510

H -1.110011 -1.320742 +0.969143

C -4.348033 +0.484000 -0.230064

H -3.218205 +0.946750 -2.026015

C -4.320793 -0.138819 +1.022819

H -3.130594 -1.275797 +2.426351

H -5.249477 +0.985665 -0.568213

H -5.199958 -0.123367 +1.658862

M_2

C -2.815445 -0.773565 +0.215703

C -1.616072 -0.071720 +0.467549

C -1.793166 +1.209214 -0.143400

N -3.584585 +0.076474 -0.495579

N -3.001236 +1.286326 -0.719647

C -4.947572 -0.140283 -0.938771

H -5.058903 -1.163738 -1.304413

H -5.656794 +0.033334 -0.122716

H -5.144645 +0.563312 -1.747643

C -0.837016 +2.354065 -0.204058

H +0.080631 +2.074029 -0.732260

H -1.306075 +3.190310 -0.728541

H -0.547105 +2.675769 +0.800777

C -3.211087 -2.153556 +0.605450

H -2.422559 -2.575399 +1.233291

H -4.148333 -2.163972 +1.172865

H -3.336381 -2.802873 -0.269106

N -0.554875 -0.622955 +1.122385

N +0.461269 +0.142898 +1.439987

C +1.643968 -0.030934 +0.766568

C +1.779334 -0.873791 -0.365823

C +2.767231 +0.713268 +1.217102

C +3.007000 -0.958740 -1.024857

H +0.920584 -1.445453 -0.701289

C +3.981893 +0.616472 +0.548197

H +2.646271 +1.350387 +2.086613

C +4.110622 -0.219916 -0.578205

H +3.104696 -1.606525 -1.891085

H +4.835979 +1.187690 +0.897069

H +5.061672 -0.293129 -1.095862

E

C +2.333869 -0.899996 +0.007844

C +1.285920 +0.034129 -0.002153

C +1.912784 +1.314111 -0.026782

N +3.468029 -0.170441 -0.019160

N +3.246880 +1.173584 -0.031621

C +4.831887 -0.654670 +0.062410

H +5.476970 +0.091735 -0.401697

H +5.134532 -0.799155 +1.104806

H +4.918488 -1.601358 -0.474992

C +1.303621 +2.676193 -0.054704

H +0.669573 +2.837923 +0.821431

H +2.099404 +3.425178 -0.071625

H +0.666438 +2.801343 -0.934617

C +2.297392 -2.389008 +0.039442

H +2.752406 -2.822229 -0.858372

H +2.823652 -2.787537 +0.913770

H +1.255427 -2.711227 +0.085903

N -0.041050 -0.380187 +0.005335

N -0.881049 +0.580931 -0.002815

C -2.236322 +0.140624 +0.000343

C -2.648857 -1.202579 -0.075695

C -3.192001 +1.163784 +0.074752

C -4.012164 -1.504636 -0.071586

H -1.901209 -1.982661 -0.138984

C -4.553978 +0.856495 +0.081665

H -2.845789 +2.190873 +0.127214

C -4.969409 -0.480976 +0.008700

H -4.332902 -2.539852 -0.132426

H -5.289657 +1.652437 +0.141848

H -6.027346 -0.723139 +0.011191

TS_iAr

C +2.689357 -0.710313 -0.011074

C +1.328254 -0.374005 -0.035191

C +1.269279 +1.045061 +0.022639

N +3.340244 +0.466827 +0.070568

N +2.511134 +1.548817 +0.082034

C +4.774614 +0.686998 +0.049104

H +5.140237 +0.763936 -0.979651

H +4.975669 +1.619521 +0.576716

H +5.278240 -0.139233 +0.554515

C +0.079268 +1.945940 +0.023554

H -0.577286 +1.742195 +0.875420

H +0.415273 +2.984471 +0.072181

H -0.524064 +1.808141 -0.879418

C +3.355636 -2.041962 -0.052375

H +4.044103 -2.120831 -0.900476

H +3.921598 -2.234672 +0.865682

H +2.590316 -2.813884 -0.152689

N +0.332628 -1.369008 -0.106694

N -0.839031 -0.964078 -0.084894

C -2.100990 -0.551587 -0.053702

C -2.719149 -0.218785 +1.186945

C -2.849623 -0.415280 -1.254119

C -4.046341 +0.207854 +1.202401

H -2.152301 -0.321316 +2.105757

C -4.165209 +0.047926 -1.197207

H -2.385785 -0.666942 -2.201983

C -4.784913 +0.357779 +0.018651

H -4.502924 +0.442173 +2.159681

H -4.713776 +0.155397 -2.129108

H -5.811407 +0.705221 +0.045383

TS_iPy

C -2.515334 -0.738234 +0.034602

C -1.225107 -0.160976 -0.173890

C -1.491593 +1.246500 -0.333359

N -3.380062 +0.284503 +0.030265

N -2.798866 +1.498010 -0.234815

C -4.820347 +0.243201 +0.207228

H -5.118339 +1.080122 +0.841840

H -5.326162 +0.324366 -0.759627

H -5.092943 -0.698792 +0.685960

C -0.519633 +2.346566 -0.602642

H +0.332579 +1.981972 -1.180336

H -1.010046 +3.153938 -1.151240

H -0.132102 +2.760608 +0.334103

C -2.850034 -2.166669 +0.276174

H -1.944476 -2.755734 +0.101161

H -3.182475 -2.347616 +1.305748

H -3.633913 -2.517202 -0.403410

N -0.090418 -0.834230 -0.199128

N +0.985498 -1.478018 -0.301403

C +2.196545 -0.709585 -0.091104

C +3.378459 -1.297577 -0.556695

C +2.238161 +0.511627 +0.598126

C +4.602809 -0.644257 -0.370857

H +3.316672 -2.255177 -1.064034

C +3.456376 +1.155992 +0.792603

H +1.313898 +0.927497 +0.983950

C +4.647731 +0.582894 +0.304127

H +5.517923 -1.092502 -0.744325

H +3.491682 +2.098248 +1.331504

H +5.596086 +1.087267 +0.456535
